# Comprehensive identification of long noncoding RNAs in colorectal cancer

**DOI:** 10.18632/oncotarget.25218

**Published:** 2018-06-12

**Authors:** Eric James de Bony, Martin Bizet, Olivier Van Grembergen, Bouchra Hassabi, Emilie Calonne, Pascale Putmans, Gianluca Bontempi, François Fuks

**Affiliations:** ^1^ Laboratory of Cancer Epigenetics, Faculty of Medicine, ULB-Cancer Research Center (U-CRC), Université Libre de Bruxelles (ULB), 1070 Brussels, Belgium; ^2^ Machine Learning Group, Computer Science Department, Université Libre de Bruxelles, 1050 Brussels, Belgium; ^3^ Inter-University Institute of Bioinformatics, Brussels, Université Libre de Bruxelles–Vrije Universiteit Brussel, 1050 Brussels, Belgium

**Keywords:** colorectal cancer, long non-coding RNAs, heterogeneity, EMT

## Abstract

Colorectal cancer (CRC) is one of the most common cancers in humans and a leading cause of cancer-related deaths worldwide. As in the case of other cancers, CRC heterogeneity leads to a wide range of clinical outcomes and complicates therapy. Over the years, multiple factors have emerged as markers of CRC heterogeneity, improving tumor classification and selection of therapeutic strategies. Understanding the molecular mechanisms underlying this heterogeneity remains a major challenge. A considerable research effort is therefore devoted to identifying additional features of colorectal tumors, in order to better understand CRC etiology and to multiply therapeutic avenues. Recently, long noncoding RNAs (lncRNAs) have emerged as important players in physiological and pathological processes, including CRC. Here we looked for lncRNAs that might contribute to the various colorectal tumor phenotypes. We thus monitored the expression of 4898 lncRNA genes across 566 CRC samples and identified 282 lncRNAs reflecting CRC heterogeneity. We then inferred potential functions of these lncRNAs. Our results highlight lncRNAs that may participate in the major processes altered in distinct CRC cases, such as WNT/β-catenin and TGF-β signaling, immunity, the epithelial-to-mesenchymal transition (EMT), and angiogenesis. For several candidates, we provide experimental evidence supporting our functional predictions that they may be involved in the cell cycle or the EMT. Overall, our work identifies lncRNAs associated with key CRC characteristics and provides insights into their respective functions. Our findings constitute a further step towards understanding the contribution of lncRNAs to CRC heterogeneity. They may open new therapeutic opportunities.

## INTRODUCTION

According to the latest statistics, colorectal cancer is the third most common cancer in men worldwide (746,000 cases, representing 10% of all cases) and the second in women (614,000 cases, 9.2% of all cases) [1]. One of the key obstacles to devising strategies for prognosis and treatment (e.g., anti-EGFR therapies) is CRC heterogeneity [2]. Nevertheless, a variety of molecular features have helped clinicians to better classify various types of colorectal tumors and scientists to better understand the molecular defects causing colorectal adenomas and carcinomas [3–5]. Such characteristics can be associated with prognosis and/or response to treatment and have been used to identify CRC subtypes [4, 6, 7]. The most commonly used molecular features include: (i) microsatellite instability (MSI) caused by a deficient DNA mismatch repair (MMR) machinery [7], (ii) chromosome instability (CIN) resulting from mutations in the APC gene [8], (iii) the CpG island methylator phenotype (CIMP), and (iv) KRAS, BRAF, and TP53 gene mutations. Unfortunately, CRC cases which may seem similar in terms of these molecular characteristics can have different outcomes or responses to treatment [2, 4, 5, 9]. This explains current efforts to discover additional features and new dimensions for describing and understanding CRC heterogeneity. The main focus of this quest has been on gene expression profiles (GEPs). These have been used, along with the above-mentioned molecular features, to distinguish up to six distinct CRC subtype [5, 10–12]. Such studies confirm how complex the etiology of CRC can be. Their findings help to explain the divergent fates of molecularly similar cases, as tumors with distinct GEPs may share similar molecular characteristics [12].

While GEPs are an invaluable resource in our effort to better understand tumor physiology, they have so far focused mostly on protein-coding mRNAs [4, 5, 12]. More recently, high-throughput sequencing has revealed the considerable size of the noncoding transcriptome [13], highlighting multiple “classes” of noncoding RNAs. One such class consists of long noncoding RNAs (lncRNAs), some of which have been shown to be functional [14, 15]. A long noncoding RNA is a transcript more than 200 bp long, which is often polyadenylated and spliced but which does not code for a protein product [16]. According to recent studies, the human genome could contain up to 48,000 lncRNA genes [17], i.e. more than twice the number of coding genes. Yet investigators have only just begun to study the functions of lncRNAs. Evidence already suggests that lncRNAs could be involved in nearly all aspects of cell function, from the regulation of pluripotency and proliferation to the etiology of diseases including cancer [15, 18–20]. Multiple teams have revealed individual lncRNAs that contribute to CRC, e. g. MALAT1, which promotes cell proliferation and migration [21], CCAT-1, which regulates chromatin conformation [22], and BANCR, which regulates EMT [23]. It remains unclear, however, in which types of CRC these lncRNAs intervene. Others have exploited lncRNA gene expression profiles to identify CRC subtypes, using them to define new tumor groups not discernable on the basis of mRNA levels [24]. These findings hint at potential contributions of lncRNAs to CRC and support the idea that long noncoding transcripts should be integrated into subtyping strategies. Much remains to be learned, however, to understand their functions and to fully assess their contribution to CRC heterogeneity.

To our knowledge, no genome-scale effort has been made to identify lncRNAs associated with colon cancer heterogeneity and to explore their potential functions. In the present study, we have used microarray data generated from 566 extensively annotated CRC samples from the Gene Expression Omnibus (GEO) public repository. To study the possible contribution of 4898 lncRNAs to pathways driving CRC diversity, we looked for transcripts whose levels correlated with the following heterogeneity and outcome markers: tumor location, genome-scale molecular alterations, oncogene mutations, mRNA-based CRC subtypes, and relapse-free survival (RFS). We then inferred functions of relevant transcripts and studied the *in vitro* consequences of depleting cells of several candidate lncRNAs in the light of their predicted functions. Overall, this work explores the lncRNA landscape across tumor diversity and investigates the potential functions of heterogeneity-associated lncRNAs. Our findings thus help create a better and more complete picture of the complex molecular networks at play in distinct CRC cases. We trust that this will pave the way to new prognostic and therapeutic opportunities.

## RESULTS

### Certain lncRNA genes are differentially regulated according to essential anatomical and genome-related characteristics of CRC

Our initial aim was to identify lncRNAs which might contribute to CRC heterogeneity. We reasoned that the corresponding genes should show particular expression patterns in relation to tumor characteristics. We thus used a large cohort of colorectal tumors and looked for lncRNA genes differentially expressed according to key molecular and anatomical features commonly used to characterize CRC [8]. We focused on tumor location (distal or proximal), CpG Island Methylator Phenotype status (CIMP positive or negative), Mismatch Repair (MMR) machinery status (deficient or proficient), and Chromosome Instability status (CIN high or CIN low), as associations have been evidenced between these features and CRC subtypes based on mRNA [12] and lncRNA [24] profiles. We stress that our aim was not to distinguish groups of tumors but to identify lncRNA genes whose expression level varies according to the presence or absence of a particular feature.

**Figure 1:**
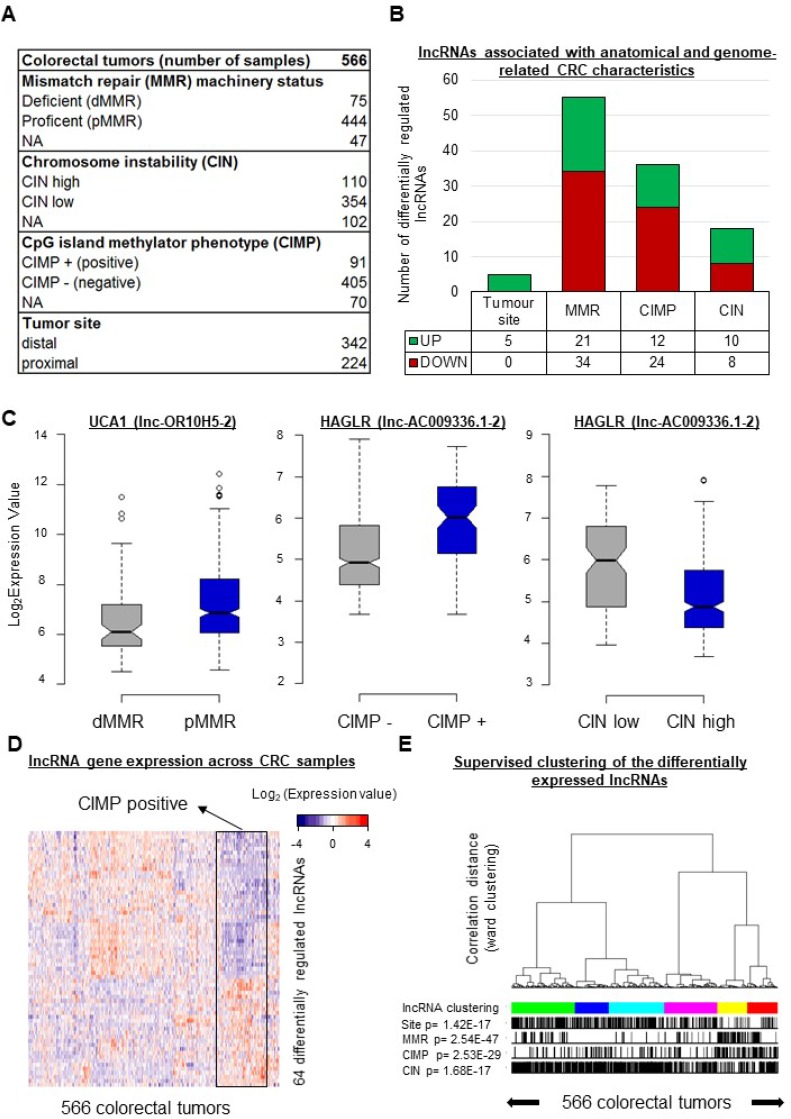
In CRC, the expression levels of 64 lncRNA genes vary according to specific tumor characteristics **(A)** Description of the colorectal cancer tissue samples analyzed in this study. (**B**) Numbers of lncRNA genes showing differential regulation (1.5 > FC < 0.67; FDR < 0.05) according to tumor location and/or MMR, CIN, and/or CIMP status. (**C**) Box plots displaying the expression levels of selected differentially regulated lncRNA genes taken as examples. UCA1 was downregulated (FC = 0.59, FDR = 0.006) in tumors with a deficient mismatch repair machinery (dMMR). HAGLR displayed the highest fold change (FC = 2.14, FDR = 3.10E-07) in CIMP-positive (CIMP+) versus CIMP-negative (CIMP−) tumors, but it was downregulated (FC = 0.46, FDR = 6.11E-08) in tumors with high chromosome instability (CIN high). (**D**) Heatmap illustrating the expression profiles of the 64 unique lncRNA genes differentially expressed according to the tumor location and/or the MMR, CIN, and/or CIMP status. (**E**) Supervised clustering of the CRC samples according to their lncRNA gene expression profiles based on the 64 genes just mentioned. *P*-values were assessed with a *t*-test and corrected for multitesting with the Benjamini-Hochberg method. Box plot description: the bold line is the median, the borders of each box are the first and third quartiles, and the whiskers (error bars) are the most extreme expression values not greater than 1.5 times the interquartile range. The notches represent the 95% confidence interval.

Array-based gene expression data were downloaded from the Gene Expression Omnibus (GEO) public repository. We selected the GSE39582 dataset for the following reasons: (i) it is, to our knowledge, the largest cohort (*n =* 566) of colorectal tumor samples with GEP data arising from the same study; (ii) each tumor has been assigned to a robust mRNA-based CRC subtype, and (iii) the dataset contains extensive clinical information, including relapse-free survival data (Figure 1A and Supplementary Table 1). All probe sets present on the Affymetrix Human Genome U133 Plus 2.0 array were reassigned in order to monitor levels of 14851 mRNAs and 4898 lncRNAs (Supplementary Table 2).

We first focused on the MMR status. Because the status of the mismatch repair machinery reflects microsatellite stability/instability, it is one of the three main molecular characteristics used to describe colorectal tumors [7, 8]. The tumors of the studied cohort were described as either MMR-deficient (dMMR) or MMR-proficient (pMMR). Upon comparing the expression levels of the 4898 lncRNA genes in pMMR and dMMR tumors, we found 55 lncRNA genes to be differentially expressed between the two groups (for dMMR versus pMMR: 0.67 > FC > 1.5, FDR < 0.05) (Figure 1B and Supplementary Table 3). According to the adopted criteria, twenty-one lncRNA genes displayed higher expression and 34 showed lower expression in the dMMR tumors. It is noteworthy that the gene corresponding to lnc-OR10H5-2 (commonly called UCA1), whose overexpression is well known in CRC tissues and associated with poorer prognosis [25], was lower in the dMMR tumors (FC = 0.59, FDR = 0.006, Figure 1C).

CIMP status (positive or negative), commonly defined on the basis of the DNA methylation patterns of the promoters of 4-8 protein-coding genes [26–28], is another major molecular feature used to characterize CRC tumors [2]. As shown in Figure 1B, we found 36 lncRNA genes to be differentially expressed in CIMP-positive versus CIMP-negative tumors. Twelve showed higher expression and 24 displayed lower expression in the CIMP-positive tumors (Figure 1B and Supplementary Table 3). Interestingly, the greatest difference between CIMP-positive and CIMP-negative tumors was observed for lnc-AC009336.1-2 (commonly called HAGLR) (Figure 1C), a lncRNA linked to neuroblastoma progression [29]. We thus compared the expression of HAGLR in tumor versus normal tissues in the colon adenoma dataset downloaded from The Cancer Genome Atlas (TCGA) database (see Materials and Methods). HAGLR was found to be downregulated in tumor samples (*n =* 155) as compared to normal tissue (*n =* 19) (Supplementary Figure 1A). Next, to examine the DNA methylation landscapes of the 36 identified “CIMP-lncRNA” genes in CRC, we downloaded and reannotated the Infinium 450 k data from the TCGA database [4] (see Materials and Methods). Of the 36 lncRNA genes differentially expressed according to CIMP status, 28 were represented on the methylation array. Of these, seven - including HAGLR - displayed differentially methylated regions in colorectal tumors as compared to normal tissue (Supplementary Figure 1B–1C and Supplementary Table 3).

The third most commonly used molecular descriptor of CRC is the CIN status [3] (high or low). We found ten lncRNA genes to show higher expression and eight to show lower expression in CIN-high than in CIN-low tumors (Figure 1B and Supplementary Table 3). Intriguingly, HAGLR appeared to be downregulated in CIN-high tumors (FC = 0.46, FDR = 6.11E-08, Figure 1C).

Only five lncRNA genes showed differential expression in distal versus proximal samples. The levels of all five transcripts were higher in distal tumors (Figure 1B and Supplementary Table 3). Although differences in molecular features and outcome have been evidenced in previous studies [7], we show here that lncRNAs are part of the molecular profile distinguishing proximal from distal tumors.

Overall, we found 64 unique lncRNA genes showing differential expression according to the tumor site or the MMR, CIMP, or CIN status. Supplementary Table 3 provides the detailed list of these lncRNAs, highlighting associations between lncRNA gene expression and CRC heterogeneity. Expression profiles limited to these 64 genes were established for the 566 studied tumors (Figure 1D). Supervised consensus clustering of the tumors on the basis of these profiles (Figure 1E and Supplementary Figure 2) revealed, as expected, associations with each of the studied features.

### TP53, BRAF, and KRAS mutations are associated with differential lncRNA expression

**Figure 2:**
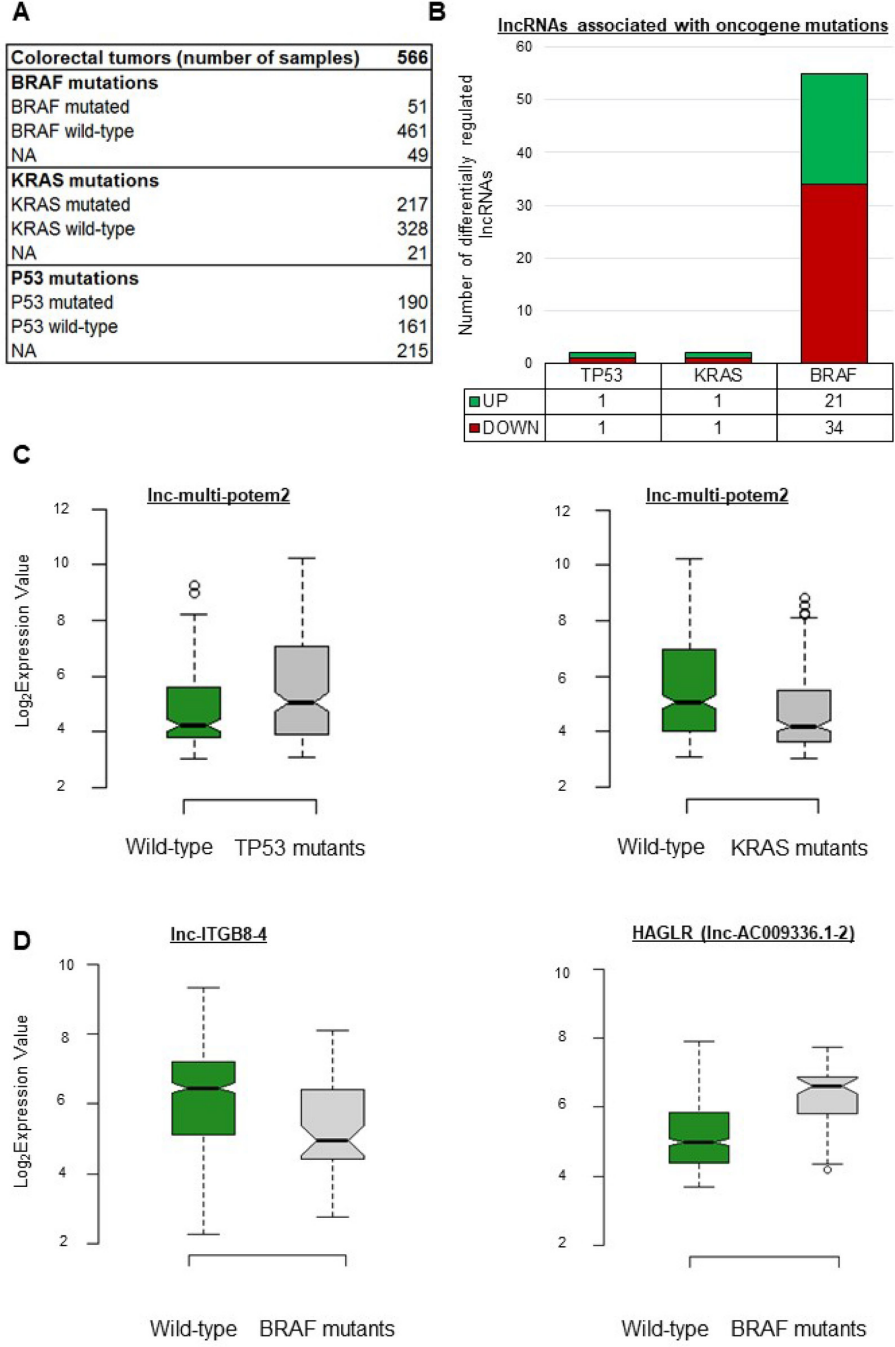
lncRNA gene expression levels depend on the oncogene mutation status in CRC (**A**) Description of the cohort as regards oncogene mutations. (**B**) Number of differentially expressed lncRNA genes per mutation. (**C**) Box plots displaying expression levels of certain lncRNA genes taken as examples. lnc-multi-potem2 was upregulated in tumors with a mutation in the TP53 gene (FC = 1.8, FDR = 0.005) but downregulated in tumors with a KRAS mutation. (**D**) In tumors with a BRAF mutation, lnc-ITGB8-4 was downregulated (FC = 0.35, FDR = 0.001) whereas HAGLR was upregulated (FC = 3.10, FDR = 2.72913E-09). *P*-values were assessed with a *t*-test and corrected for multitesting with the Benjamini-Hochberg method. Box plot description: the bold line is the median, the borders of the box are the first and third quartiles, and the whiskers (error bars) are the most extreme expression values not greater than 1.5 times the interquartile range. The notches represent the 95% confidence interval.

Previous studies having revealed the existence of a BRAF-activated lncRNA involved in CRC [23] and of a p53-regulated lncRNA [30], we reasoned that oncogenic mutations might influence or be associated with the expression levels of certain lncRNA genes. The data available for our cohort included mutation information for TP53, KRAS, and BRAF (Figure 2A). As shown in Figure 2B, only two lncRNA genes showed differential expression in tumors with a TP53 or KRAS mutation as compared to wild-type tumors. Interestingly, lnc-multi-POTEM-2 appeared upregulated in TP53-mutant tumors but downregulated in KRAS-mutant tumors (Figure 2C and Supplementary Table 3). Fifty-five lncRNAs showed differential regulation in BRAF-mutant as compared to wild-type tumors (Figure 2B and Supplementary Table 3). When a BRAF mutation was present, lnc-ITGB8-4 showed the strongest downregulation and HAGLR, the strongest upregulation (Figure 2D).

Of the 4898 lncRNA genes whose expression we monitored, 59 showed significantly higher or lower expression in tumors harboring a mutation in BRAF, KRAS, or TP53 (Supplementary Table 3). Our results thus reveal new lncRNAs which may be affected by these mutations commonly found in CRC. We believe that additional mutation-status-associated lncRNAs will emerge as relevant data become available.

### lncRNA levels vary across CRC subtypes

**Figure 3:**
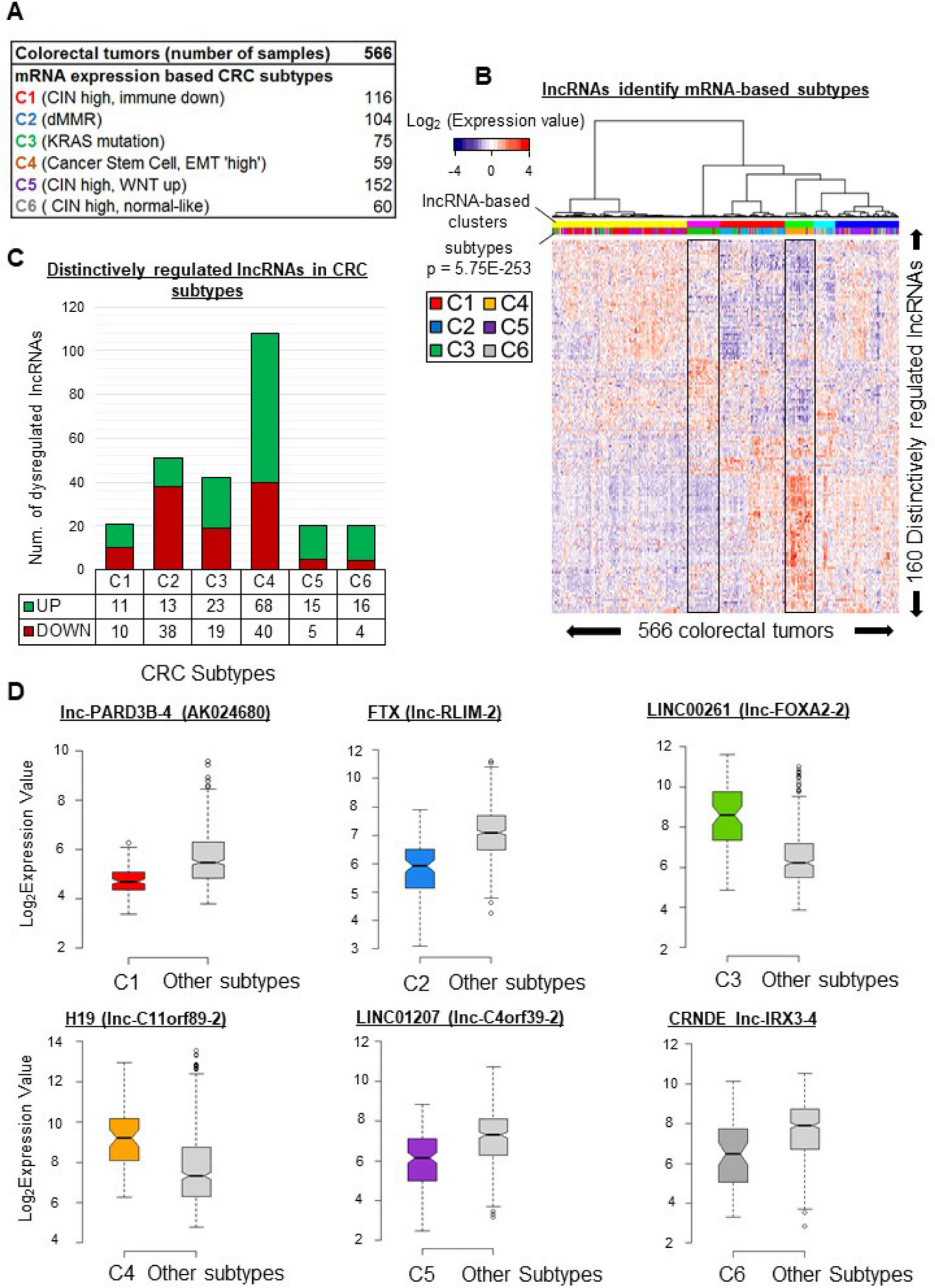
lncRNA gene expression patterns vary across CRC subtypes (**A**) Distribution of six mRNA-profile-based CRC subtypes [12] in the studied cohort. (**B**) Supervised clustering of tumors based on their lncRNA gene expression profiles. The profiles were based on levels of 160 unique lncRNAs identified as distinctively regulated in at least one subtype. (**C**) Number of distinctively regulated lncRNAs in each colorectal cancer subtype. The average expression level of an lncRNA in a given subtype is compared to its average level in all remaining subtypes. An lncRNA was considered to be distinctively regulated in a subtype when the fold change was above 1.5 or below 0.67 (FDR < 0.05) (**D**) Box plots illustrating the distinctive regulation of exemplative lncRNAs in given subtype. lnc-PARD3B-4 (also identified as AK024680), was downregulated (FC = 0.6, FDR = 8.7E-25) in C1-subtype tumors. lnc-RLIM-2 (also identified as FTX), was downregulated (FC = 0.44, FDR = 8.12E-21) in C2-subtype tumors. lnc-FOXA2-2 (also identified as LINC00261), appeared upregulated (FC = 5.18, FDR = 2.14E-14) in C3-subtype tumors as compared to all the other subtypes. H19 (identified here as lnc-C11orf89-2), was upregulated (FC = 3.6, FDR = 1.2E-06) in the C4 subtype. LINC01207 (identified here as lnc-C4orf39-2), appeared downregulated (FC = 0.45, FDR = 4.09E-11) in the C5 subtype. lnc-IRX3-4 (also identified as CRNDE), was downregulated (FC = 0.4, FDR = 0.0001) in the C6 subtype. *P*-values were assessed with *t*-test and corrected for multitesting with the Benjamini-Hochberg method. Box plot description: the bold line is the median, the borders of the box are the first and third quartiles and the whiskers (error bars) are the most extreme expression values not greater than 1.5 times the interquartile range. The notches represent the 95% confidence interval.

Large cohorts of heterogeneous tumors can be classified into subtypes based on both mRNA expression profiles and the above-mentioned clinical characteristics [12]. These subtypes can in turn be studied individually to reveal specificities useful in selecting appropriate treatment solutions [5, 9, 19, 31]. Pursuing our search for lncRNAs which may contribute to tumor diversity, we sought to identify transcripts displaying subtype-associated patterns of expression. On the basis of mRNA expression profiles, Marisa *et al.* have described six CRC subtypes (Figure 3A and Supplementary Table 1) associated with molecular and clinical characteristics as well as disease outcome [12]. To find lncRNA genes distinctively expressed in a particular subtype, we compared the mean expression level of each of the 4898 lncRNA genes in each subtype with the mean of the means of the other subtypes (because of this ‘one versus all others’ approach, we use below the term “distinctive expression” rather than “differential expression” to describe the results). We identified 160 unique lncRNA genes showing particularly high or low expression in at least one subtype. Clustering of the tumors according to these 160 unique lncRNA gene expression profiles revealed six significant clusters (Figures 3B and Supplementary Figure 3), but only two of these appeared almost exclusively enriched in a single subtype (see the boxed profiles corresponding to the magenta cluster, rich in C3, and the green cluster, rich in C4).

The lncRNAs showing up- or downregulation in each subtype are detailed below.

### C1 subtype

As shown in Figure 3C, 21 lncRNA genes displayed distinctive expression (11 high and 10 low) in the C1 subtype. Among the downregulated transcripts, lnc-PARD3B-4 (also called AK024680) has been associated previously with disease outcome [32].

### C2 subtype

In this subtype, 38 lncRNAs were downregulated and 13 were upregulated (Figure 3C). The lncRNA FTX (identified here as lnc-RLIM-2), linked to regulation of the XIST noncoding transcript [33], was downregulated (FC = 0.44, FDR = 8.12E-21, Figure 3D).

### C3 subtype

In this subtype, 19 lncRNAs were downregulated and 23 were upregulated (Figure 3C). In particular, LINC00261 (lnc-FOXA2-2), involved in EMT regulation [34], was upregulated (FC = 5.18, FDR = 2.14E-14, Figure 3D).

### C4 subtype

We found 108 distinctively regulated lncRNA genes, 40 downregulated and 68 upregulated (Figure 3C). We notably found H19 (lnc-C11orf89-2) and lnc-FAM172A-2 to be upregulated in C4 samples (FC = 3.63 and FC = 1.6, respectively, FDR < 0.001, Figure 3D and Supplementary Figure 4A).

### C5 subtype

Our analysis associated 20 lncRNA genes with the C5 subtype (5 downregulated, 15 upregulated genes) (Figure 3C). In this subtype, we observed downregulation of LINC01207 (identified here as lnc-C4orf39-2), which promotes cell proliferation in lung adenocarcinoma [35] (FC = 0.452816992, FDR = 4.09E-11, Figure 3D).

### C6 subtype

In the C6 subtype, 20 lncRNA genes showed distinctive expression. The levels of four lncRNAs were lower in this subtype than in any other, while another 16 lncRNAs were upregulated (Figure 3C). Interestingly, CRNDE (FC = 0.4, FDR = 0.0001, Figure 3D) appeared downregulated, whereas it was upregulated in subtype-C5 tumors (Supplementary Table 3).

CRC subtypes have been defined on the basis of mRNA profiles. Specific mRNA expression profiles have revealed driving pathways in each set of tumors [12] whereas the analysis of distinctive lncRNA gene expression in these subtypes potentially extends the pools of molecules contributing to these diverse tumor phenotypes. We believe that such transcripts might represent a new set of diagnostic or prognostic markers or therapeutic targets.

### lncRNAs are associated with relapse-free survival in CRC

**Figure 4:**
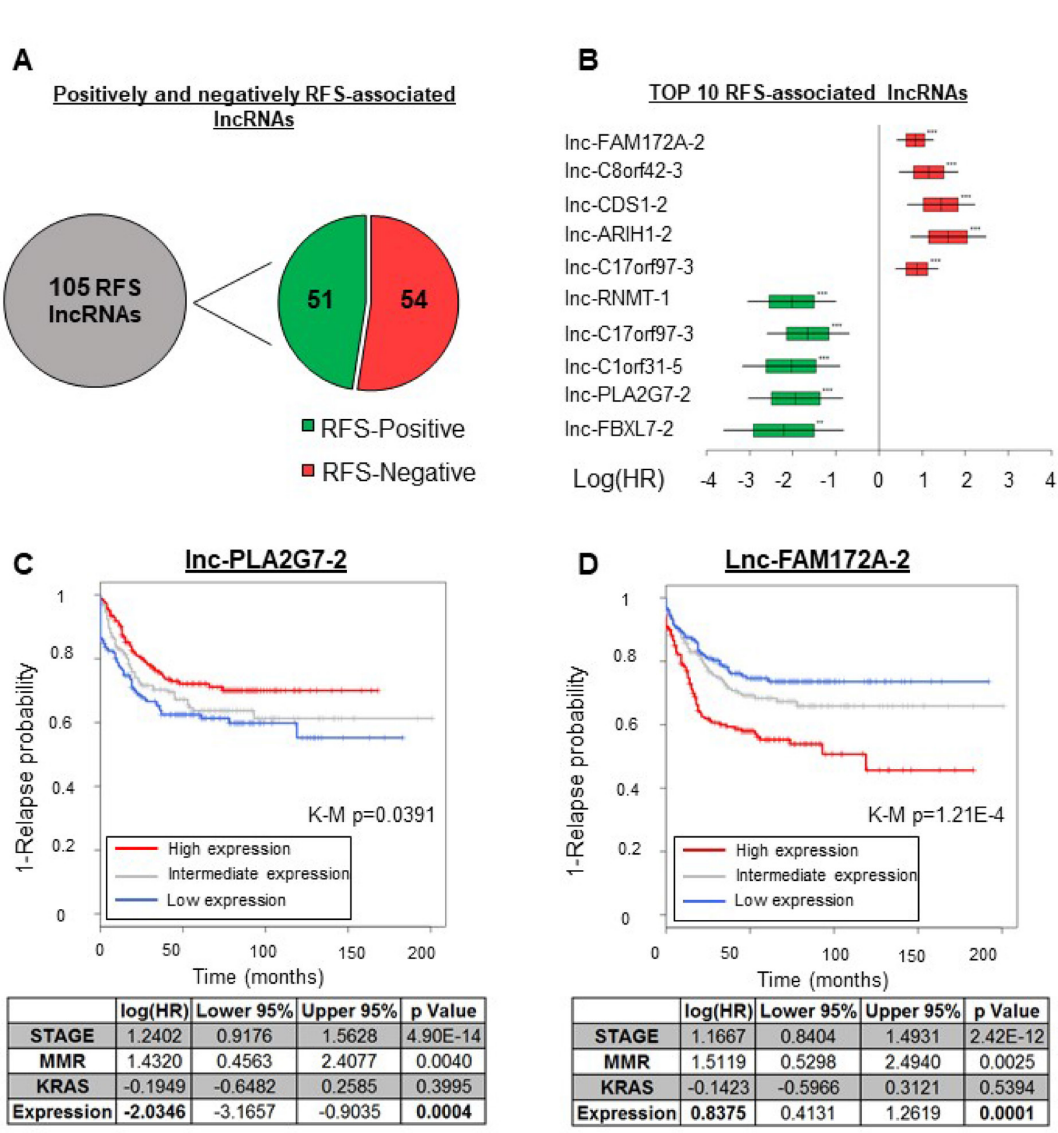
lncRNAs associated with the relapse-free survival time in CRC Uni- and multivariate Cox regression analyses revealed 105 lncRNAs to be associated with RFS (Supplementary Table 3). (**A**) Numbers of lncRNAs positively and negatively associated with the RFS time. (**B**) Forest plot showing the log2 HR values with SDs (boxes) and 95% confidence intervals (bars). The data are from the relapse-free survival analysis (multivariate Cox analysis) of the five most significantly RFS-associated lncRNAs. The data for positively RFS-associated lncRNAS are in green and the data for negatively RFS-associated ones are in red. (**C**)Top: Kaplan–Meier curve illustrating the univariate association of lnc-PLA2G7-2 with RFS. Bottom: Multivariate analysis. (**D**)Top: Kaplan–Meier curve illustrating the univariate association of lnc-FAM172A-2 with RFS. Bottom: Multivariate analysis. K-M p = Kaplan–Meier curve associated *p*-value.

RFS is another parameter capturing tumor heterogeneity. We thus used both uni- and multivariate analyses to seek associations between lncRNA gene expression profiles and RFS. We performed a Cox regression analysis for each of the 4898 lncRNAs, using the array and RFS data pertaining to the GSE39582 cohort. In our multivariate analysis we included the mismatch repair machinery status, the KRAS mutation status, and the disease stage as covariables, chosen for their strong association with RFS in the univariate Cox regression analyses (Supplementary Table 6). We did not limit our analyses to a specific time window but used the full extent of the RFS data available (see Materials and Methods). As shown in Figure 4A, we found in both the uni- and multivariate analyses a significant association between the expression levels of 105 lncRNA genes and relapse-free survival time (Hazard ratio (HR) < 0.67, HR>1.5; *p <* 0.05). The list of these genes is supplied in Supplementary Table 3. The observed correlation was positive for 51 of these genes and negative for 54 of them (Figure 4A–4B), and in what follows the corresponding lncRNAs are respectively called ‘RFS-positive’ and ‘RFS-negative’ lncRNAs. Hazard ratios (HR) across all 105 lncRNAs ranged from 9.3 to 0.2 (Figure 4B and Supplementary Table 3). Among the RFS-positive lncRNAs, lnc-PLA2G7-2 was the most significantly associated with RFS in the univariate analysis (HR = 0.27, *p =* 0.000835), remaining a top predictor in the multivariate analysis (HR = 0.25, *p =* 0.000485). Figure 4C shows a Kaplan–Meier curve for the association of lnc-PLA2G7-2 with RFS, where patients with high expression of this lncRNA (red curve) display a lower relapse tendency. Figure 4D, on the other hand, shows a Kaplan–Meier curve for lnc-FAM172A-2, which was negatively associated with RFS. This effect was most significant in the univariate analysis (HR = 1.9, *p =* 1.4E-06) but FAM172A-2 remained a top predictor in the multivariate analysis (HR = 1.84, *p =* 3.31E-05) (Figure 4B and Supplementary Tables 6–7).

In all, we identified 282 unique long noncoding genes on the basis of associations with tumor location, genome-scale molecular features, oncogene mutations, CRC subtypes, or RFS. To gain further insight into their potential contribution to CRC heterogeneity, the next step was to investigate their potential functional roles in colorectal cancer.

### Feature- and subtype-related lncRNAs are associated with key CRC-related processes

We used guilt-by-association (GbA) analysis [36] (see Materials and Methods) to gain insights into potential functions of the 282 identified lncRNAs. Briefly, we computed a correlation matrix for each of these lncRNAs and the protein-coding mRNAs corresponding to 70 gene sets. The gene sets spanned five major aspects of tumor biology (cell adhesion, metabolism, cell cycle, immunity, and signaling pathways) and other relevant biological processes such as the epithelial-to-mesenchymal transition and angiogenesis (Supplementary Tables 8 and 9). We then used the correlation coefficients to generate ranked lists of mRNAs for each of the 282 lncRNAs and subjected these lists to Gene Set Enrichment Analysis (GSEA) [37, 38]. This yielded Normalized Enrichment Scores (NESs) associating each of the 282 selected lncRNAs with each of the 70 gene sets (Supplementary Figure 5). Then all NESs pertaining to lncRNAs of the same group, i.e., lncRNAs associated with the same clinical parameter (CIMP status, CRC subtype, RFS…), were aggregated to obtain an Enrichment Metascore (EM) describing the relationship between a given group of lncRNAs and a particular gene set. We then focused on significant (FDR < 0.05) EMs. This provided insights into potential functions of lncRNAs associated with the studied markers of tumor heterogeneity.

**Figure 5:**
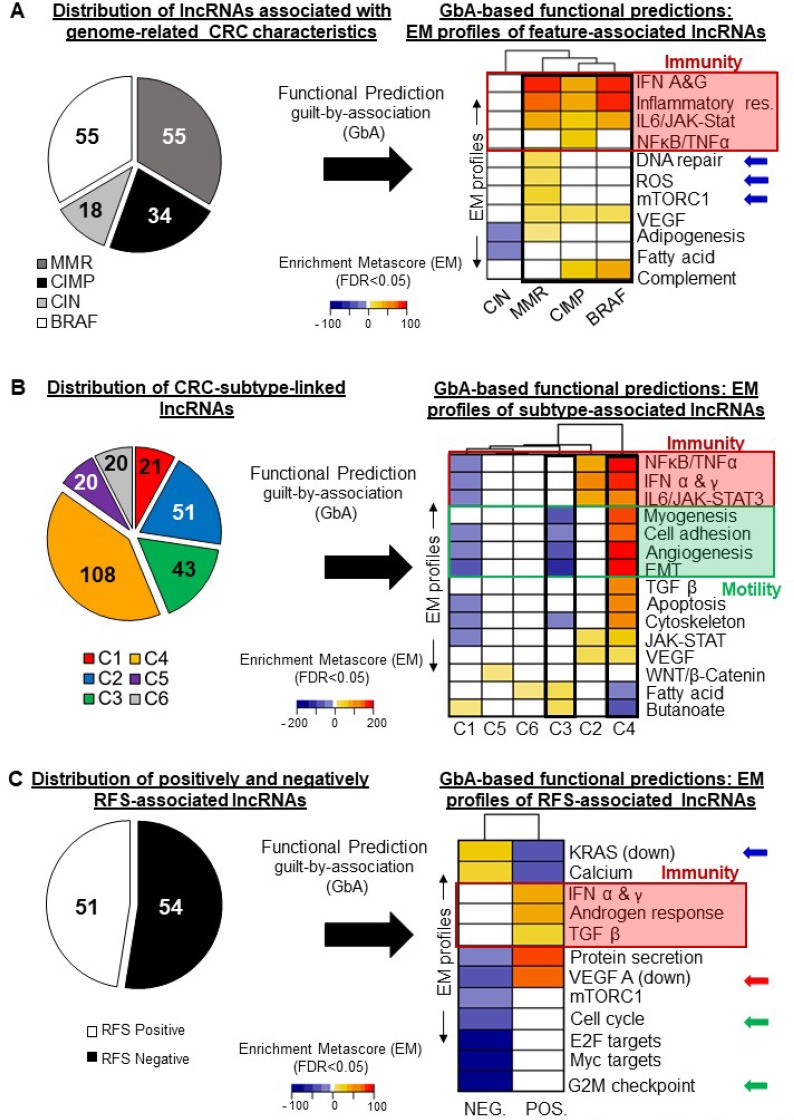
Guilt-by-association analysis: functional predictions for selected lncRNAs Heatmaps illustrating the pathways whose activation (orange to red tones) or inhibition (blue tones) correlates with the expression of clinical-parameter-associated lncRNA genes. To relate each group of lncRNAs to gene sets, an enrichment meta-score (EM) was computed. For example, a high EM was computed for CIMP-associated lncRNAs and the NFκB/TNFα gene set, indicating a tight correlation between CIMP-associated lncRNA-gene expression levels and those of genes involved in the NFκB and TNFα signaling pathways (5A, right panel, red frame). (**A**) Left: numbers of lncRNAs differentially regulated according to the CIMP, MMR, CIN, or BRAF status. Right: EM profiles of the lncRNAs associated with each genomic alteration, reflecting their specific and shared associations with gene sets. (**B**) Left: Numbers of lncRNAs distinctively regulated in each of the six CRC subtypes described by Marisa *et al.* (C1 to C6). Right: Heatmap showing the EM profiles of the lncRNAs associated with each subtype, reflecting their specific and common associations with gene sets. (**C**) Left: Numbers of lncRNAs positively (RFS-Pos) or negatively (RFS-Neg) associated with relapse-free survival. Right: Heatmap showing the EM profiles of RFS-associated lncRNAs. Blue, yellow and red tones reflect significant (FDR *<* 0.05) associations with gene sets. White rectangles represent no significant association.

No significant EMs were observed for the lncRNAs associated with tumor location or with a KRAS or P53 mutation. Figure 5A shows the numbers of lncRNAs associated with the MMR, CIMP, CIN, and BRAF statuses (left panel) and a heatmap of their respective EM profiles, describing their relation to gene sets (right panel). MMR-, CIMP-, and BRAF-associated lncRNAs shared rather similar Enrichment Metascore profiles, quite different from that of the CIN-associated lncRNAs. In the following sections we describe the putative functions of lncRNAs associated with key CRC characteristics.

### MMR-associated lncRNAs

This was the only group showing a significant association with the DNA repair gene set (EM = 22.1, FDR < 0.01) (Figure 5A, right panel, blue arrows). We observed a unique association between MMR-associated lncRNAs and the reactive oxygen species (ROS) (EM = 16.8, FDR < 0.001) and mTORC1 gene sets (EM = 32.6, FDR < 0.05) (Figure 5A, right panel, blue arrows). It is noteworthy that this group was also strongly and positively associated with the inflammation (EM = 74.85, FDR < 0.05), interferon α/γ (EM = 87.97, FDR < 0.01), and IL6/JAK-STAT3 (EM = 57.91, FDR < 0.05) gene sets (Figure 5A, red frame). These results show that the lncRNAs showing differential regulation in dMMR versus pMMR tumors might influence interactions with components of the immune system.

### CIMP-associated lncRNAs

as seen in Figure 5A, this group also exhibited positive associations with the inflammation (EM = 67.35, FDR < 0.001), interferon α/γ (EM = 68.01, FDR < 0.001), and IL6/JAK-STAT3 (EM = 44.40, FDR < 0.01) gene sets (red frame). CIMP-associated lncRNAs, however, were distinguished by their association with the NFκB/TNFα (EM = 53.80, FDR < 0.05) gene set.

### CIN-associated lncRNAs

had a very distinct EM profile. They only showed negative EMs, for adipogenesis (EM = −8.01, FDR < 0.05) and for fatty acid metabolism (EM = −9.93, FDR < 0.05) (Figure 5A, right panel). Our data thus highlight a unique relationship between CIN-positive tumors and adipogenesis, possibly mediated by lncRNAs.

### BRAF-mutation-associated lncRNAs

Among the highest and most significant EMs exhibited by BRAF-associated lncRNAs were those corresponding to the inflammation (EM = 91.26, FDR < 0.001), VEGF (EM = 18.83, FDR < 0.001), and IL6/JAK-STAT3 (EM = 59.94, FDR = 0.03) gene sets (Figure 5A, right panel, red frame).

Overall, and apart from the above-mentioned specific associations (MMR-associated lncRNAs and DNA repair, CIMP-associated lncRNAs and NFκB/TNFα), we observed strong associations with immune-system-related gene sets and a general association with the VEGF signaling pathway for all groups of lncRNAs except CIN-associated lncRNAs.

Next, to extend our view of the molecular networks involved in CRC heterogeneity, we proceeded as described above to generate EM profiles for the lncRNAs associated with each CRC subtype and to infer potential functions. In Figure 5B, the left panel shows the number of lncRNAs distinctively regulated in each subtype and the right panel shows the clearly distinctive EM profile of each subtype. These profiles are described below. All lncRNAs associated with CRC subtypes are listed in Supplementary Table 3.

### C1-associated lncRNAs

These lncRNAs notably displayed negative EMs for the NFκB/TNFα (EM = –39.4, FDR < 0.05), IFN α & *γ* (EM = −41.34, FDR < 0.01), and IL6/JAK-STAT3 (EM = –35.74, FDR < 0.001) gene sets (red frame), corresponding to essential immunity-related pathways. C1-associated lncRNAs also exhibited negative correlations with the angiogenesis (EM = –32.36, FDR < 0.01) and EMT (EM = –55.55, FDR < 0.05) gene sets (green frame).

### C2-associated lncRNAs

These lncRNAs showed positive EMs for all gene sets negatively associated with the C1-associated group: C2-associated lncRNA levels were found to correlate tightly with those of mRNAs involved in the NFκB/TNFα and IL6/JAK-STAT3 pathways (Figure 5B, right panel, red frame). As C2-subtype tumors exhibit a highly active immune system signature [12], our results suggest that lncRNAs identified here may contribute to the different immune response behaviors of these two subtypes.

### C3-associated lncRNAs

This group exhibited the most negative EMs for motility-related gene sets, i.e. the cell adhesion (EM = –43.14, FDR < 0.05), myogenesis (EM = –54.81, FDR < 0.01), angiogenesis (EM = –64.3, FDR < 0.001), and EMT (EM = –115.4, FDR < 0.001) gene sets. These results tally with previous observations describing this subtype as having a weak mRNA-based motility signature [12].

### C4-associated lncRNAs

This subtype differs strongly from the C3 subtype in terms of clinical outcome [12]. C4-associated and C3-associated lncRNAs displayed opposite EM profiles (Figure 5B, right panel, black frames), since the C4-lncRNAs had the most positive EMs for the cell adhesion (EM = 158.89, FDR < 0.001), myogenesis (EM = 174.50, FDR < 0.001), angiogenesis (EM = 190.94, FDR < 0.001), and EMT (EM = 340.01, FDR < 0.001) (Figure 5B, right panel, green frame) gene sets. These associations highlight the importance of the EMT-angiogenesis axis in CRC etiology, while suggesting it may involve an lncRNA component.

### C5-associated lncRNAs

This group appeared associated only with the WNT/β-catenin signaling data set, in a positive manner (EM = 6.64, FDR < 0.05) (Figure 5B, right panel). This constitutes additional evidence that this pathway is particularly dominant in these tumors [5, 12]. It is noteworthy that CRNDE, which appears upregulated in this subtype (Supplementary Figure 4B), has recently been demonstrated to promote renal carcinoma cell proliferation via the WNT/β-catenin pathway [39].

### C6-associated lncRNAs

The lncRNAs showing distinctive regulation in the C6 subtype appeared associated only with the fatty acid metabolism gene set (EM = 12.45, FDR < 0.05) (Figure 5B, right panel).

The above analyses highlight important potential functions for subtype-associated lncRNA genes, as their expression levels correlate tightly with those of well-described sets of coding genes. These findings give additional support to the essential nature of some pathways or processes (EMT, angiogenesis, IL6/JAK-STAT3, WNT/β-catenin) in different types of CRC, while adding a new molecular dimension to their networks.

### Relapse-associated lncRNAs

To gain insight into the potential roles of the 105 lncRNAs identified here as associated with RFS (see Figure 4), we separated them into two categories: those negatively associated (RFS-negative) and those positively associated (RFS-positive) with survival (Figure 5C, left panel). We then generated Enrichment Metascores (EMs) for each category separately. The right panel of Figure 5C shows a heat map of the most significant EMs associated with these two groups of lncRNAs. As expected, we observed a striking difference in EM directionality. We found the RFS-negative lncRNAs to correlate positively with the KRAS Signaling Down gene set (EM = 48.56, FDR < 0.001) and the RFS-positive lncRNAs to correlate negatively with it (EM = –62.03, FDR < 0.001) (Figure 5C, right panel, blue arrow). The two groups of lncRNAs also showed opposite associations with the genes downregulated by VEGF A activation (red arrow). This again highlights the importance of this axis in CRC. We additionally found a negative correlation between RFS-negative lncRNAs and gene sets involved in cell cycle regulation (Cell cycle EM = –55.13, FDR < 0.01 and G2M Checkpoint EM −93.60, FDR < 0.001), while RFS-positive lncRNAs showed no significant correlation with these gene sets (Figure 5C, right panel, green arrows). RFS-positive lncRNAs did display a positive correlation with the following immune-system-related gene sets: TGF-β (EM = 40.01, FDR < 0.05), IFNα & *γ* (EM = 59.06, FDR < 0.01), and androgen response (EM = 56.45, FDR < 0.001) (Figure 5C, right panel, red frame). These observations highlight the potential contribution of lncRNAs to key pathways known to contribute to CRC outcome [4, 5]. Furthermore, the evidenced correlations between RFS-associated lncRNAs and less described pathways suggest new possible roles for these pathways in the context of CRC.

Overall, these results give lncRNAs a place in important processes related to colorectal cancer and shed new light on their involvement in the various avenues leading to this complex disease. Our findings provide a basis for further experimental investigation of lncRNA-related mechanisms. Such studies may yield new therapeutic opportunities for colorectal and other cancers.

### Functional characterization of identified lncRNAs:

Having used computational approaches to generate our functional hypothesis regarding the 282 heterogeneity-associated lncRNAs, we then selected three lncRNAs for further *in vitro* functional characterization and to bring experimental support to their potential functions inferred from the GbA analysis. We selected lnc-BLID-5, lnc-GNB4-1 and lnc-AKAP3-1 for the following reasons: (i) the first two transcripts displayed upregulation in the C4 “EMT-active” subtype and direct correlation with the EMT and cell migration gene sets, while lnc-AKAP3-1 was selected for its upregulation in the C3 “EMT-weak” subtype and its negative correlation with the EMT gene set (Supplementary Figure 6A and 6C), (ii) ZEB1, a master regulator of EMT [9], was among the top mRNAs whose levels correlate with the expression levels of lnc-BLID-5 and lnc-GNB4-1 (Supplementary Figure 6B) and (iii) lnc-BLID-5 and lnc-GNB4-1 correlated negatively with the cell cycle gene set, but only lnc-GNB4-1 showed a significant correlation. lnc-AKAP3-1 displayed no significant association with this gene set (Supplementary Figure 6C).

To assess the function(s) of these lncRNAs, we performed RNA-interference-mediated knockdown in the following colon carcinoma cells: HCT-116, RKO, and HT-29 (see Material and Methods). These cell lines were selected as they possess distinct molecular features [40]. It is worth noting that HCT-116 and RKO cells display microsatellite instability (dMMR) and are both CIMP positive but CIN negative. These cell lines also share a BRAF V600E mutation while remaining of the wild type for TP53 and KRAS [40]. The HT-29 cell line, on the other hand, presents microsatellite stability (pMMR) and is both CIMP and CIN positive. In this cell line, the KRAS oncogene allele is of the wild type but both BRAF and TP53 are mutated. Another important feature considered when selecting the CRC cell lines was their EMT activity. Loboda *et al.,* who have developed an EMT activity signature, describe the HCT-116 and RKO cells as EMT “active” and HT-29 cells as “not active” [10].

### Candidate lncRNAs affect cell proliferation

**Figure 6:**
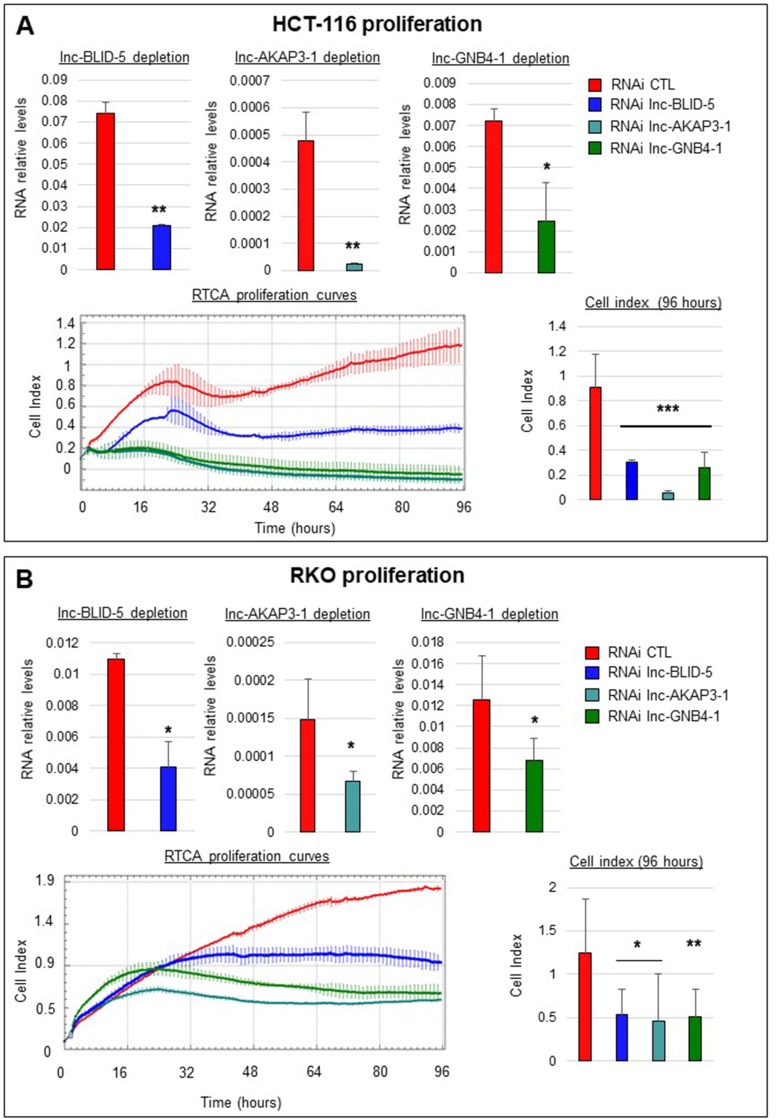

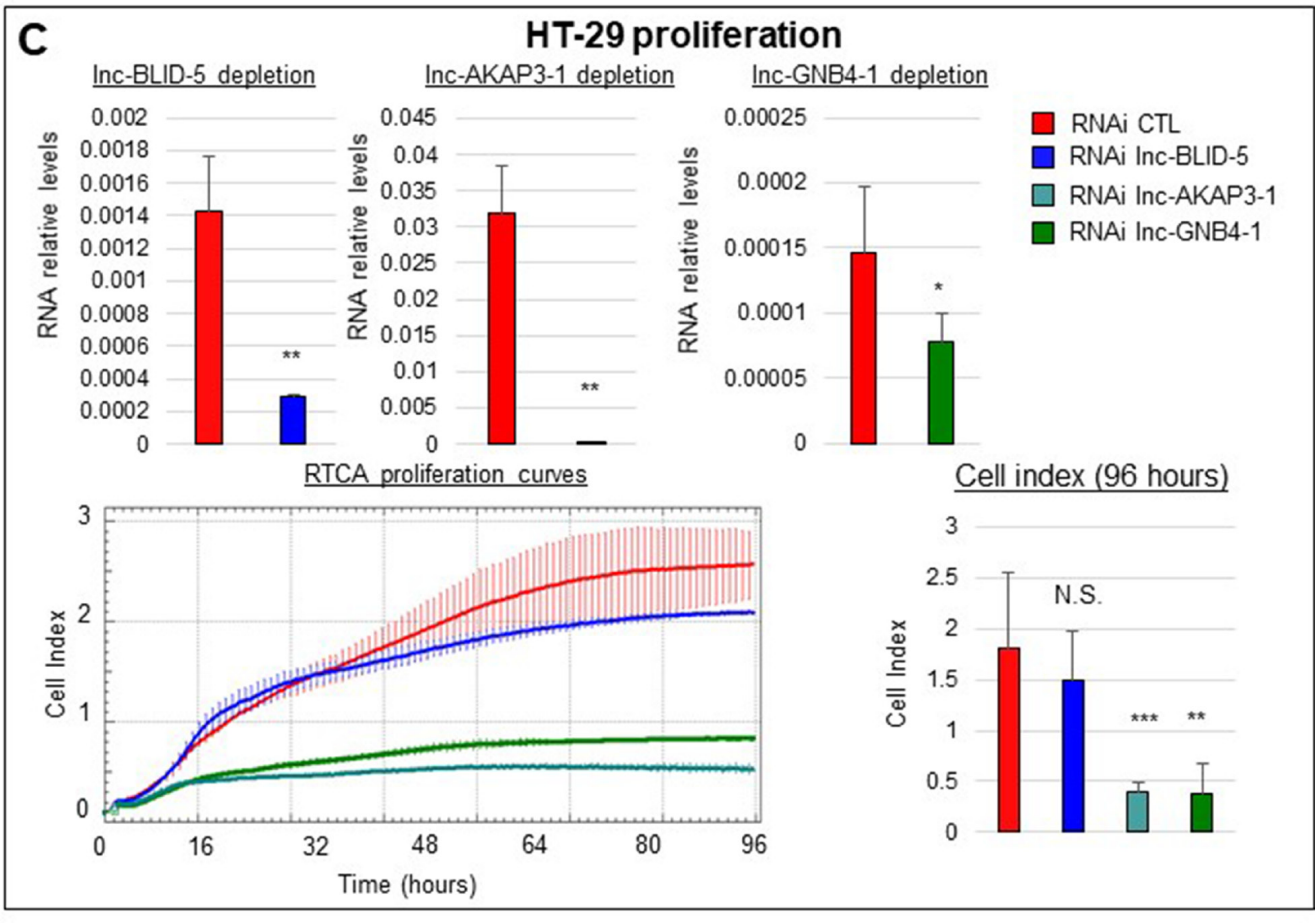
Selected lncRNA candidates affect cell proliferation (**A**) Top panel: from left to right: RNA interference mediated knockdown of lnc-BLID-5, lnc-AKAP3-1, and lnc-GNB4-1 in HCT-116 cells. Error bars represent standard deviations of three biological replicates. Bottom left panel: real-time proliferation curves of HCT-116 cells treated with the CTL RNAi (red) or an RNAi targeting lnc-BLID-5 (blue), lnc-AKAP3-1 (dark cyan), or lnc-GNB4-1 (dark green). A representative experiment is shown for at least three biological replicates. Error bars represent standard deviations of four technical replicates. Bottom right panel: average cell indexes after 96 hours, errors bars represent standard deviations of three biological replicates. (**C** and **B**) Same as A for RKO and HT-29 cells. Significant differences were evaluated with a two-tailed paired *t*-test (N.S. = not significant, *p* < 0.05 = ^*^, *p* < 0.01 = ^**^, *p* < 0.001 = ^***^).

To test the influence of our candidate lncRNAs on cell proliferation, we used xCELLigence technology to assess real time proliferation. The top panels of Figure 6A illustrate the average levels of depletion obtained with siRNA for each candidate lncRNA (see Materials and Methods). The bottom left panel of Figure 6A depicts the proliferation curves of HCT-116 cells treated with siRNAs against lnc-BLID-5 (blue curve), lnc-AKAP3-1 (dark cyan curve), and lnc-GNB4-1 (dark green curve) for 96 hours. After 96 hours, cells treated with the control siRNA reached an average cellular index of 1.2 (Figure 6A, bottom right panel), whereas cells depleted of lnc-BLID-5 transcripts were impaired in their proliferation and only reached an average cell index of 0.3 (Figure 6A, bottom right panel). The impact of lnc-AKAP3-1 depletion on cell proliferation appeared greater than that of lnc-BLID-5, as cells depleted of this transcript never reached an average cell index greater than 0.05 after 96 hours (Figure 6A, bottom right panel). Moreover, during the proliferation assays, the density of cells treated with an siRNA against lnc-AKAP3-1 decreased, possibly reflecting cell death and loss of adherence. When lnc-GNB4-1 was depleted in HCT-116 cells, these cells reached an average cell index of 0.26 (Figure 6A, dark green curve) and also showed signs of cell death, as the final cell index was inferior to the initial one (in at least one experiment). Average levels of expression reached after siRNA treatment of RKO cells are depicted at the top of Figure 6B. Depletion of lnc-BLID-5 in RKO cells also impaired cell proliferation (Figure 6B, bottom left panel, blue curve): cells treated with an siRNA against this transcript reached an average cell index of only 0.53 after 96 hours, whereas control cells averaged at 1.24 (Figure 6B, bottom right panel). We also found cells depleted of lnc-GNB4-1 and lnc-AKAP3-1 to display diminished proliferation, as after 96 hours they reached cell indexes of 0.46 and 0.51 respectively (Figure 6B, bottom right panel). The top panels of Figure 6C illustrate the achieved depletion levels in HT-29 cells. These cells retained their ability to proliferate when treated with lnc-BLID-5 siRNA (Figure 6C, bottom left panel, blue curve), but depletion of both lnc-AKAP3-1 and lnc-GNB4-1 led to strikingly decreased proliferation (Figure 6C, bottom left panel, dark cyan and dark green curves respectively): HT-29 cells treated with the CTL siRNA reached a cell index of 1.8, while cells with reduced levels of lnc-AKAP3-1 and lnc-GNB4-1 appeared unable to reach cell indexes greater than 0.5.

### Candidate lncRNAs affect cell migration

**Figure 7:**
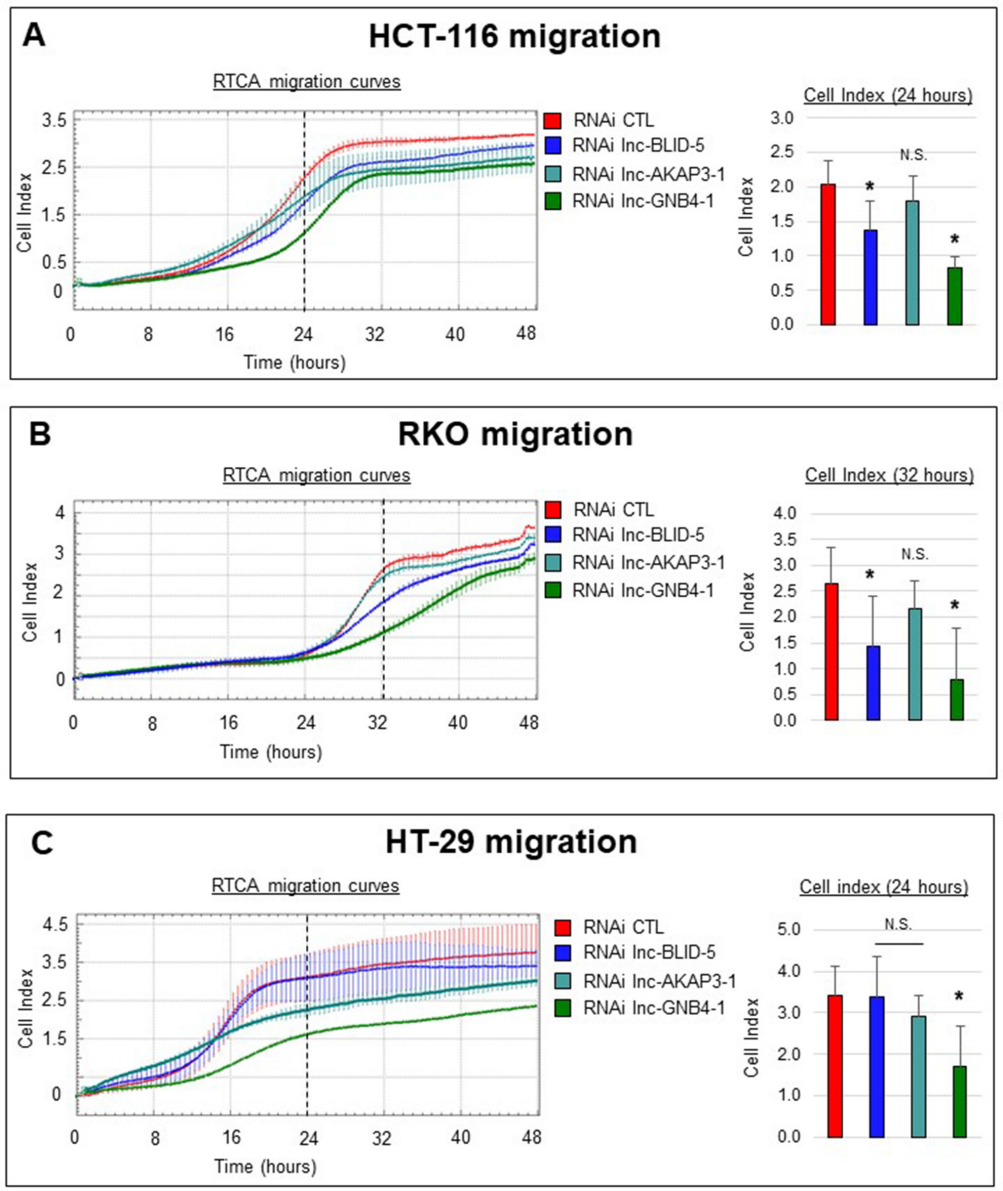
Selected lncRNA candidates affect cell migration (**A**) Left panel: real-time migration curves of HCT-116 cells treated with the CTL RNAi (red) or RNAi targeting lnc-BLID-5 (blue), lnc-AKAP3-1 (dark cyan) or lnc-GNB4-1 (dark green) in the presence of 20% FBS used as chemoattractant. A representative experiment is shown for at least three biological replicate. Error bars represent the standard deviation of four technical replicates in the. Right panel: average cell indexes after 24 hours, errors bars represent standard deviation of three biological replicates. (**B**) Left panel: real-time migration curves of HCT-116 cells treated with the CTL RNAi (red) or RNAi targeting lnc-BLID-5 (blue), lnc-AKAP3-1 (dark cyan) or lnc-GNB4-1 (dark green) in the presence of 20% FBS used as chemoattractant. A representative experiment is shown for at least three biological replicate. Error bars represent the standard deviation of four technical replicates in the. Right panel: average cell indexes after 32 hours, errors bars represent standard deviation of three biological replicates. (**C**) Same as A for HT-29 cells. Significant differences were evaluated with a two-tailed paired *t*-test (N.S. = not significant, *p* < 0.05 = ^*^, *p* < 0.01 = ^**^, *p* < 0.001 = ^***^).

We then evaluated the effects of candidate knockdowns on migration, a process closely linked to ECM interactions and EMT [41] (the corresponding gene sets being directly associated with lnc-BLID-5 and lnc-GNB4-1 and indirectly associated with lnc-AKAP3-1, Supplementary Figure 6C). For this we used the xCELLigence system again, this time with fetal bovine serum (FBS) as a chemoattractant placed in the lower chamber. We monitored cell migration for 48 hours. The left panels of Figure 7 illustrate the migration curves of cells (HCT-116, RKO, and HT-29) treated with CTL siRNA (red curve) or with an siRNA targeting lnc-BLID-5 (blue curve), lnc-AKAP3-1 (dark cyan curve), or lnc-GNB4-1 (dark green curve). Migration of HCT-116 cells occurred after sixteen hours and appeared finished after 32 hours. After 24 hours, control cells displayed an average cell index (CI) of 2, while the CI reached by cells depleted of lnc-BLID-5 transcripts was only 1.4 (Figure 7A, right panel). At the same time point, cells treated with an siRNA against lnc-GNB4-1 reached an average CI of 0.8. These observations suggest a positive influence of both of these lncRNAs on cell migration. In contrast, cells in which lnc-AKAP3-1 had been knocked down appeared unchallenged in their ability to migrate. Compared to HCT-116, RKO cells displayed an eight-hour delay in the initiation of migration, as most of this process occurred between 24 and 40 hours. In this cell line, lnc-BLID-5 and lnc-GNB4-1 again appeared necessary for proper cell migration, as their depletion caused the average CI reached after 32 hours to decrease significantly (Figure 7B, right panel). As in HCT-116 cells, lnc-AKAP3-1 depletion did not impair migration of RKO cells (Figure 7B, right panel). In the HT-29 cell line, only lnc-GNB4-1 depletion significantly affected migration. After 24 hours, control cells reached a CI of 3.4, whereas cells depleted of lnc-GNB4-1 reached an average CI of only 1.6 (Figure 7C, right panel).

**Figure 8:**
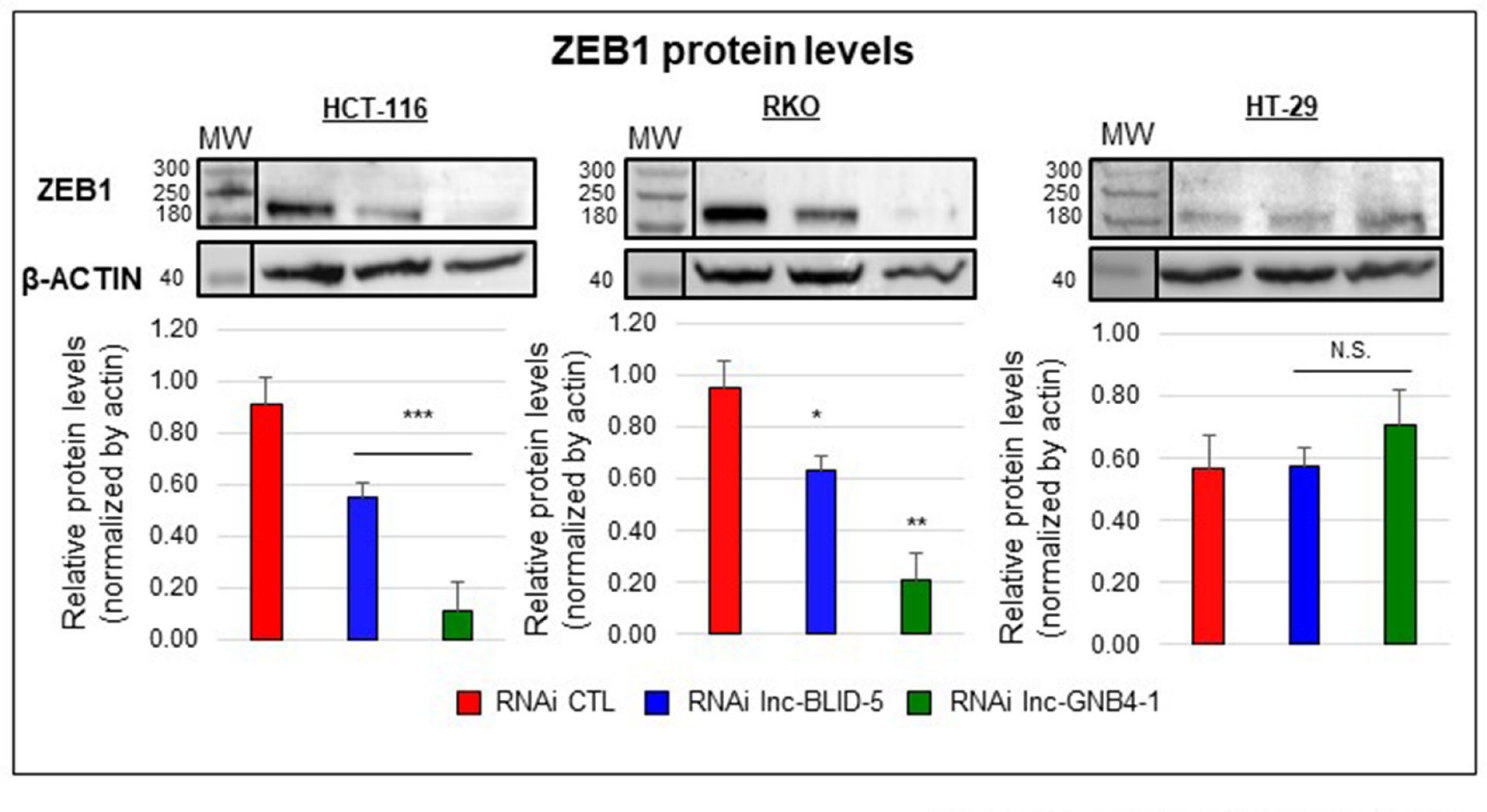
The EMT-associated transcripts lnc-BLID-5 and lnc-GNB4-1 regulate the ZEB1 protein level Top panel: Western blot analysis of ZEB1 and β-ACTIN protein levels in cells treated with the CTL RNAi (red) or an RNAi targeting lnc-BLID-5 (blue) or lnc-GNB4-1 (dark green). A representative experiment is shown for three biological replicates. Bottom panel: average relative protein levels. The ZEB1 band intensity was quantified with the ImageJ software and normalized to the intensity of the β-ACTIN band. Errors bars represent standard deviations of three biological replicates. Significant differences were evaluated with a two-tailed paired *t*-test (N.S. = not significant, *p* < 0.05 = ^*^, *p* < 0.01 = ^**^, *p* < 0.001 = ^***^).

Because the levels of lnc-BLID-5 and lnc-GNB4-1 correlated with those of ZEB1 mRNAs (Supplementary Figure 6D), we assessed the impact of their depletion on ZEB1 protein levels. In HCT-116 and RKO cells, both lnc-BLID-5 and lnc-GNB4-1 caused the ZEB1 protein level to drop, by ∼80% and ∼40% respectively (Figure 8, left and middle panels). In HT-29 cells, the basal level of ZEB1 protein appeared lower than in HCT-116 and RKO cells (Figure 8, right), and so was the corresponding mRNA level (Supplementary Figure 8). Nevertheless, No significant reduction of the ZEB1 protein level was observed in HT-29 cells depleted of either lnc-BLID-5 or lnc-GNB4-1 (Figure 8, right panel).

Overall, these experiments support our *in silico* predictions showing that (i) all three candidate lncRNAs are required for proper cell proliferation, (ii) both transcripts directly associated with the EMT gene set (lnc-BLID-5 and lnc-GNB4-1) appear to favor cell migration, and (iii) both depletion of either of these transcripts results in a reduced level of ZEB1 protein, a transcription factor essential to the regulation of the EMT [9, 42]. While we have focused on proliferation, migration, and EMT for illustrative purposes, these results lend weight to the other associations uncovered in this study. We believe our findings should facilitate further research into the roles played by lncRNAs in the various types of CRC, thus, most likely, expanding the range of treatment and prognosis options.

## DISCUSSION

CRC heterogeneity is a major problem in the treatment of this cancer. It complicates the choice of a therapeutic strategy, leads to diverse drug responses, and makes the development of new targeted therapies more complex [2, 5, 9]. Recent efforts to assess CRC subtypes have improved our comprehension of the molecular networks at play in the main types of CRC, but we are far from understanding all molecules contributing to CRC development and heterogeneity. This leaves pools of potential therapeutic targets untapped. Although lncRNAs have been used to classify CRC tumors [24] and although several functional and mechanistic studies have extended our understanding of individual lncRNAs [43, 44], little is known about the functions of lncRNAs in particular types of CRC.

To identify lncRNAs potentially contributing to distinct CRC phenotypes, we first looked for lncRNAs displaying expression patterns associated with key features of colorectal tumors: location, MMR status, CIN status, CIMP status, mutational status (TP53, BRAF, and KRAS), and subtype. This first screen yielded 282 unique lncRNA genes. We then used a guilt-by-association (GbA) analysis to generate hypotheses on the function(s) of these transcripts. The results of our GbA analysis point to the involvement of lncRNAs in the pathways distinctively altered in CRC subtypes. The key findings of this analysis are summarized and discussed below.

First, our computational approach has generated several associations corroborated by previous studies. For instance, our analysis suggests a link between UCA1 (lnc-OR1OH5-2) and the cell cycle (cf its correlation with the Cell Cycle and G2M Checkpoint gene sets, Supplementary Table 8). Accordingly, multiple studies have demonstrated both *in vitro* and *in vivo* the influence of UCA1 on proliferation [45, 46]. Likewise, we have found H19 to be most strongly associated with the EMT gene set (Supplementary Table 8), in agreement with recent work describing the mechanism by which H19 promotes EMT in colorectal cancer through miRNA sequestration [47]. Finally, according to our GbA analysis, only one group of lncRNAs appears associated with the WNT/β-catenin signaling pathway: those showing distinctive regulation in the C5 subtype. This finding is in line with previous work describing subtype C5 as having a strong WNT pathway activation signature [12]. More importantly, our results indicate that lncRNAs may participate in dysregulation of this pathway. We believe that these observations further support the reliability of our GbA approach, thus strengthening other associations revealed by our study.

A second point worth discussing is the link between lncRNAs and both EMT and angiogenesis. This link has been mentioned previously [23, 48], and there is evidence that EMT promotes the expression of proangiogenic factors and increases tumor angiogenesis [49]. EMT is also widely described as a crucial process in colorectal cancer [9, 10, 41]. Our data show that both C3-associated and C4-associated lncRNAs are associated with EMT and angiogenesis, but in opposite ways. This suggests that these lncRNAs might be new molecular players involved in these CRC-outcome-influencing processes. A study by Chen *et al.* has demonstrated that it is possible to discriminate tumors on the basis of lncRNA gene expression [24]. These investigators identified 229 lncRNAs and succeeded in clustering tumors into five clinically relevant subtypes on the basis of their levels. Our reannotation method has enabled us to monitor the expression of 67 of these 229 lncRNAs. We have found only nine of them to be associated with heterogeneity markers: eight with the C4 subtype and one with dMMR tumors (Supplementary Table 3). These findings again highlight the peculiarity of the C4 subtype as regards lncRNAs, suggesting that these molecules may participate in EMT, the driving process of this subtype [5, 12]. The poor overlap between our set of lncRNAs reflective of CRC heterogeneity and the set of lncRNAs identified by Chen *et al.*, suggests that other factors may influence lncRNA-gene transcription in CRC. The fully non-supervised clustering approach followed by Chen *et al.* might have identified transcripts whose levels are indicative of particular tumors but are not associated with the molecular features studied here. Third, we have found RFS-positive and RFS-negative lncRNAs to show opposite associations with the VEGF-A gene set (composed of genes repressed by VEGF-A activation) and we have observed a tight correlation of RFS-positive lncRNAs with genes of the TGF-β pathway. The potential involvement of lncRNAs in these pathways might be worthy of further investigation, since VEGF and TGF-β inhibitors are among the few drugs shown to increase patient survival [50, 51]. Hence, RFS-associated lncRNAs might represent a new angle from which to target these pathways. Another reason for targeting lncRNAs is that developing an antisense oligonucleotide that block production of a protein should be easier than designing protein-targeting compounds [52]. Previously, Hu *et al.* identified a signature predictive of CRC relapse, composed of six lncRNAs [32]. We therefore tested the prognostic value of this signature in our multivariate analysis and likewise found it to be associated with RFS (Supplementary Table 7). We failed, however, to find one of the lncRNAs of this signature, CR622106, in either the Ensembl or the REF-Seq reference transcriptome, and no single lncRNA in this group proved predictive of relapse. Furthermore, we found three of the lncRNAs composing the signature - lnc-ITGBL1-2 (also called AK026784), lnc-PARD3B-4 (AK024680), and lnc-RP1-239B22.1.1-1 (AK123657) - to show distinctive regulation in the C4 and C1 subtypes. Interestingly, we evidenced a positive association between lnc-ITGBL1-2 and lnc-PARD3B-4, both associated with shorter RFS, and the EMT and angiogenesis gene sets. On the other hand, lnc-RP1-239B22.1.1-1, linked to longer RFS, had a negative association with these gene sets (Supplementary Table 8). Our GbA analysis thus offers new insights into the roles of certain RFS-associated lncRNAs in CRC. Overall, the 105 RFS-associated lncRNAs identified here by both uni- and multi-variate analysis may represent a new pool of prognostic markers liable to be used individually or in combination to identify patients with a low risk of relapse and who could thus be excluded from aggressive therapies.

To get some idea of the reliability of our computer-based functional predictions, we have performed a series of loss-of-function studies describing the influence of three candidate lncRNAs on cell proliferation and migration. Lnc-BLID-5 depletion resulted in impaired proliferation in both the HCT-116 and RKO cell lines yet did not affect HT-29 cells. This cell line’s apparent indifference to Lnc-BLID-5 depletion may be due to the low level of this transcript, as HT-29 cells displayed a lower basal level of lnc-BLID-5 (Supplementary Figure 7A) than either HCT-116 or RKO cells. Silencing lnc-AKAP3-1 or lnc-GNB4-1 resulted in significant inhibition of cell proliferation in all three cell lines. While both lnc-BLID-5 and lnc-GNB4-1 displayed a significant (and negative) association with the cell cycle gene set, lnc-AKAP3-1 did not (Supplementary Figure 6C). lnc-AKAP3-1 did, on the other hand, display a negative correlation with the EMT gene set, although it did not significantly influence cell migration. lnc-GNB4-1 and lnc-BLID-5 correlated positively with EMT and appeared to favor cell migration. These observations may be due to the difficulty of translating *in vivo* observations to *in vitro* models. Indeed, despite sharing similar molecular characteristics a cell line cannot perfectly represent a population of tumors and cannot behave as tumor cells within a tumor and its micro-environment. Moreover, transcriptomic correlation observed in the RNA of biopsies could reflect distinct cellular composition and be the result of adipocytes or lymphocytes infiltration which vary across colorectal subtypes. This said, the role of lnc-GNB4-1 and lnc-BLID-5 in the regulation of migration and EMT was further supported by our findings that their depletion is accompanied by a loss of ZEB1 protein levels in HCT-116 and RKO cells. Various mechanisms might explain the effect of lnc-BLID-5 and lnc-GNB4-1 on ZEB1 protein production. For instance, transcripts could serve as precursors for miRNAs involved in the repression of ZEB1 mRNAs as is the case with the miR-200 family of miRNAs [53, 54]. Alternatively, these transcripts may recruit transcription factors required for ZEB1 transcription or interfere with epigenetic repressive agents, as described before for certain lncRNAs [55]. Further investigation will be necessary to understand how lnc-BLID-5 and lnc-GNB4-1 regulate ZEB1. The knowledge gained could result in the development of new ways to target EMT in CRC. Of note, depletion of lnc-GNB4-1 impaired migration of HT-29 cells but did not result in lower levels of ZEB1 proteins, suggesting that other factors mediate this transcript’s control over migration.

Finally, the present study does have its limitations. Firstly, our microarray approach is restricted to detecting lncRNAs targeted by probe sets present on the Affymetrix U133 Plus array, which represent only a fraction of the lncRNAs encoded by the human genome (up to ∼10% of certain databases) [17]. Nevertheless, our study has generated important insights and advances, by providing a detailed list of lncRNAs associated with key tumor characteristics, along with potential function(s). Moreover, the clinical information available for our cohort was more extensive than the clinical annotation of the TCGA cohort, especially as regards heterogeneity markers and median follow-up (3.58 years for the cohort used here, versus 2.17 years for the TCGA microarray cohort and 2.25 years for the TCGA RNA-Seq cohort). Secondly, our study differs from previous work in that we have focused on tumor tissues only. This contrasts, for instance, with the work of Chen *et al.*, who by comparing lncRNA gene expression profiles of lncRNAs between normal tissue, primary tumors, and metastasis identified lncRNAs associated with metastasis and thus prognostic of disease progression [56]. One should note, however, that of the 282 lncRNAs identified here, 209 were also present on the Agilent G4502A microarray used in the TCGA cohort and 61 of these have been found to be dysregulated in cancers as compared to normal tissues (Supplementary Figure 9 and Supplementary Tables 3, 4). This suggests an involvement of the 61 transcripts in the development of CRC, while highlighting the fact our approach has captured CRC-heterogeneity-associated lncRNAs that would have been overlooked had we focused on lncRNAs dysregulated in tumors as compared to normal tissue.

In conclusion, the present in-depth analysis of the lncRNA transcriptome in colorectal cancer has identified 282 lncRNAs reflecting the heterogeneity of this cancer. We have further predicted potential functions of these lncRNAs, showing that they may be at play in major pathways/processes relevant to CRC, and most importantly in the TGF-β and WNT pathways, immunity, EMT, and angiogenesis [5, 9, 10, 12]. Results supporting the predicted functions of lnc-BLID-5, lnc-GNB4-1 and lnc-AKAP3-1 were obtained in several experiments, arguing in favor of the overall effectiveness of our integrated approach. We therefore believe our work (i) will expand our view of the molecular networks composing the above-mentioned essential pathways so as to include lncRNAs and (ii) will highlight the prevalence of these axes in the different types of CRC. This should facilitate further research that may lead to exploiting lncRNAs as prognostic markers or therapeutic targets for customized treatment of CRC.

## MATERIALS AND METHODS

### Colorectal cancer gene expression data and Affymetrix microarray reannotation

We downloaded from the GEO database gene expression data from the microarray study of Marisa *et al.* (U133 Plus 2.0 Affymetrix) (accession number: GSE39582). The raw CEL files were frma normalized in the R environment, using the limma and frma packages to obtain a log_2_ normalized expression signal for each probe set. We then applied the combat algorithm from the sva library with default parameters to adjust the data for batch effects. The probe sets were locally mapped by sequence alignment (NCBI BLAST 2.2.29+) against a reference transcriptome composed of the ENSEMBL 74 transcriptome (excluding long noncoding transcripts) and the LNCIPEDIA database V3, dedicated to lncRNAs [57]. For lncRNA profiling in tumor samples, we required that at least 80% of a probe set target a transcript of the LNCipedia database and discarded probes with discordant transcript biotype information between LNCipedia and Ensembl.

We also excluded probe sets having multiple targets, unless a target lncRNA arose from a duplicated region of the genome. To identify duplicate lncRNAs, we blasted all lncRNA transcripts against the LNCipedia database, and two transcripts with different names were defined as duplicates if the shorter transcript shared at least 95% of its sequence with the longer one. Since duplicate lncRNAs could not be distinguished from each other, they were considered to correspond to a single gene whose name was generated by adding the tag ‘multi’ to the name of one of the duplicate lncRNAs. Alternative transcripts were viewed as only one lncRNA arising from the largest locus determined by transcript mapping. When multiple probe sets were assigned to the same gene, the one with the highest variance across samples was selected. We provide a full annotation table for the 4898 lncRNAs, including each one’s genomic location, category, nearest coding genes, and the probe sets matching it (Supplementary Table 2). We believe providing this information will facilitate further comparisons of our results with those of other studies. Most importantly, this detailed reannotation should facilitate the use of microarray data obtained with U133 Plus 2.0 Affymetrix, a widespread platform, for the analysis of lncRNAs in other contexts.

### CRC primary tumor and normal tissue gene expression data from TCGA and Agilent microarray reannotation

We downloaded colon adenocarcinoma (COAD) gene expression profiles from TCGA. Raw data were processed as previously described [4]. Probes of the TCGA microarray were mapped to the LNCipedia database, using the TCGA annotation file. Briefly, coordinates targeted by TCGA microarray (Agilent 4502A) probes were first extracted from the annotation file available at the TCGA website and converted to the hg19 genome build. Then probes where at least 58 bp of the targeted region overlapped, in a strand-specific way, with exons of lncRNA transcripts in the LNCipedia V3 database were selected. Because the boundaries of the exons are not always clearly defined, we added 5 bp on both sides of each lncRNA exon.

### DNA methylation data (Infinium 450 k) from TCGA and Illumina Infinium 450 k array reannotation

The Infinium 450k array was annotated for lncRNAs by intersecting targeted cytosine positions on the Human hg19 genome with long noncoding transcript positions from LNCipedia v3. A probe was defined as located in the promoter of an lncRNA gene if the targeted cytosine was located –2 kb to +1 kb from the TSS. It was defined as located in the gene body if it was located anywhere else between the TSS and the TTS. The same approach was used with transcripts from GENCODE v19, RefSeq v58, and UCSC (downloaded in 2013) to annotate probes for coding and small noncoding transcripts. TCGA 450k colon adenocarcinoma data were downloaded (in March 2015) as raw data from the TCGA Data Portal and pre-processed with in-house scripts. Briefly, raw Infinium data were filtered by removing low-quality data, using a detection *p*-value threshold of 0.05. Cross-reactive probes (i.e. targeting several genomic locations) and probes containing SNPs were filtered out, using the extended annotation provided by Price *et al.* [58] (see Dedeurwaerder *et al.* [59] for a detailed description). Probes associated with X and Y chromosomes were removed from the analysis. β-values were computed with the formula: β-value = M/[U+M], where M and U are the raw “methylated” and “unmethylated” signals, respectively. The β-values were corrected for type I and type II bias by peak-based correction [59, 60]. Cytosines differentially methylated between normal tissues and cancers were identified according to the recommendations in [59]. First the methylation values were converted to M-values with the following formula: M-value = log_2_(β-value/(1 – β-value)). The statistical significance of differential methylation was assessed with a t-test applied to these M-values. In parallel, a delta-β was computed as the absolute difference between the median β-value within each category (cancer versus normal). Cytosines showing a *p*-value < 0.05 together with an absolute delta-β > 0.2 were reported as differentially methylated.

### Clinical data and molecular subtype prediction

Clinical data were downloaded from GEO (Supplementary Table 5). Tumor location and the MMR, CIMP, and CIN statuses were determined as described in the original studies. CRC subtypes were determined on the basis of sample mRNA expression profiles as described by Marisa *et al.* [12].

### Identification of lncRNAs associated with particular anatomical or genome-scale molecular features or with oncogene mutational status

To identify noncoding transcripts relevant to colorectal cancer biology, we focused on transcripts associated with available clinical parameters. We selected transcripts differentially expressed between samples with different tumor locations or different MMR, CIN, or CIMP statuses. We also selected lncRNA genes showing differential expression between samples having or not a mutation in the BRAF, KRAS, or P53 gene. Fold change had to be above 1.5 or below 0.67, with FDR < 0.05.

### Identification of lncRNA genes showing distinctive expression in CRC subtypes

We identified lncRNA genes distinctively expressed in one particular CRC subtype as compared to all the others combined. For this we compared the mean expression level of each lncRNA gene in a given subtype to the mean of the means calculated for the remaining subtypes. Fold change had to be above 1.5 or below 0.67, with FDR < 0.05.

### Identification of lncRNAs associated with clinical outcome

To find lncRNAs that might be associated with RFS, we performed both univariate and multivariate Cox regression analyses, using the “survival” library in R. We included the KRAS mutational status, MMR machinery status, and disease stage in the multivariate analyses as covariables, because they appeared significantly associated with RFS in a univariate Cox regression analysis. Proportional hazard assumptions were tested with the “cox.zph” function (threshold 0.01). In all analyses, an lncRNA was considered associated with RFS if it satisfied the following criteria: (i) the corresponding hazard ratio was above 1.5 or below 0.67; (ii) the *p*-value was equal to or smaller than 0.05.

### Functional predictions for lncRNAs by guilt-by-association analysis

GbA analysis was used to correlate expression levels of the selected lncRNA genes with those of 70 sets of protein-coding genes known to be involved in particular functions. We computed the correlations and generated a ranked list of mRNAs for each lncRNA. This list was then subjected to GSEA. The gene sets were obtained from the KEGG (Kyoto Encyclopedia of Genes and Genomes) database and the Molecular Signatures Database (MsigDB). The 70 gene sets used in the GSEA covered five major aspects of tumor biology (cell adhesion, metabolism, cell cycle, immunity, and signaling pathways) and other biological processes considered to be relevant in the current context. A complete list of the gene sets used is supplied in Supplementary Table 9. In accordance with GSEA software guidelines, we grouped gene sets containing redundant genes as follows: (i) we computed a distance matrix between gene sets using the overlap distance (defined as the number of common genes divided by the number of genes composing the smallest gene set), (ii) we performed hierarchical clustering based on this matrix (complete linkage), (iii) we used a threshold of 0.5 to cut the tree and grouped the gene sets belonging to the same cluster. GbA analysis enabled us to generate hypotheses regarding the functions of given lncRNAs. We chose to focus only on the 282 lncRNAs identified here because GbA analysis is computationally demanding. First, we divided our colorectal tumor expression data into two datasets, each corresponding to 283 samples. In the two groups of samples the CRC subtype distribution was the same, but otherwise allocation to the groups was random. For each dataset, we computed a Pearson correlation matrix linking each lncRNA to each coding gene, thus producing two matrices of 282 lncRNAs x 9,675 mRNAs. We then ranked the mRNAs in each matrix on the basis of their coefficients of correlation to a given lncRNA. The Gene Set Enrichment Analysis (GSEA) software (parameters: 1000 permutations on gene sets, min size = 15, max size = 500) was then used to calculate an enrichment score for each gene set on the basis of the relative ranks of members and non-members of the investigated gene set. We thus obtained two matrices containing an enrichment score and an FWER statistic for each “lncRNA/gene set” pair (282 lncRNAs x 70 gene sets). To obtain a high-confidence association of lncRNAs with functions, we finally selected gene sets that were statistically (FWER < 0.05) associated with an lncRNA in both matrices, and computed the mean of their enrichment scores.

To link sets of subtype- or tumor-feature-associated lncRNAs with particular functions/gene sets, we computed for each identified set of lncRNAs an enrichment metascore (EM) defined as the weighted sum of the enrichment scores obtained for each lncRNA member in the set, the attributed weight being -1 if the lncRNA appeared downregulated and 1 otherwise. Then 10,000 random groups of lncRNAs of the same size as the lncRNA set of interest were generated by random selection. The same weighted sum approach was used to calculate a metascore for each of these groups. The *p*-value of the metascore was defined as the proportion of randomly generated metascores that were at least as high (low) as the metascore of the positively (negatively) associated set.

### Culture of the CRC cell lines and target lncRNA genes silencing

HCT116 and HT-29 cells were grown at 37° C under 5% CO2 in McCoy’s medium (Gibco) supplemented with 10% FBS (Gibco). RKO cells were grown at 37° C under 5% CO2 in Eagle Minimum Essential Media (Sigma) supplemented with 10% FBS (Gibco). To silence the target lncRNA gene, we used locked nucleic acid (LNA) Gapmers^®^ (Exiqon) to induce RNA interference silencing. Briefly, cells were transfected in a 6-well plate (or an xCELLigence 16-well plate) with 35 nM LNA Gapmer and 5 µl lipofectamine 2000^®^ (Invitrogen) in 3 ml total volume (or 200 µl in the case of 16-well plates) and incubated for 48 hours before collection for qPCR and western blot analysis. In the case of proliferation/migration assays cells were transfected 40 minutes after being seeded. LNA Gapmer sequences: Negative control (A), 5′-AACACGTCTATACGC-3′; lnc-BLID-5, 5′-CGATGACTCGACAATC-3′; lnc-GNB4-1, 5′-AATCGCTGAGGTCATA-3′; lnc-AKAP3-1, 5′-CGTGCTTCCGGTGATA-3′.

### RNA purification and real-time quantitative PCR

RNA purification was performed with the RNAeasy kit (Qiagen) according to the manufacturer’s instructions. DNase treatment was performed with a DNA-free DNase kit (Ambion) according to the manufacturer’s protocol. Quantitative PCRs were performed with SYBR Green dye (Eurogentec) in a LightCycler 480 (Roche). Briefly, cDNA was reverse-transcribed from 1 μg RNA with random hexamers (Amersham/Pharmacia Biotech) and Superscript II reverse transcriptase (Life Technologies, Inc.). Results were normalized with respect to the housekeeping genes SDHA, GAPDH, and HPRT1. qPCR assay primer sequences: lnc-BLID-5, 5′-TGCGTGTTTCCAAAGTGAGG-3′ (forward), 5′-AAGCCAGCATCATCGGTAGT-3′ (reverse). Lnc-AKAP3-1, 5′- GCCAAGAACTTCGGAAGCAT-3′ (forward), 5′- GGGTCAGTCTGAGGGATGTT-3′ (reverse). Lnc-GNB4-1, 5′- GGATCACGAGGTCAGGAGTT-3′ (forward), 5′- CCTCACGAGTAGCTGACAGG-3′ (reverse). ZEB1, 5′-TGAATGCGAGTCAGATGCAG-3′ (forward), 5′-CTCTTCAGGTGCCTCAGGAA-3′ (reverse).

### Cell proliferation/migration

To evaluate the proliferation capacity, cells were seeded in wells. Given their different proliferation rates, HCT-116 was seeded at 8000 cells/well, RKO cells at 20000 cells/well, and HT-29 at 12000 cells/well in an xCELLigence E-plate 16 (Roche). After 40 minutes of stabilization, the cells were transfected with an LNA Gapmer directed against a given lncRNA candidate. The instrument derives a cell index from the electric impedance and gives a real-time representation of the growth characteristics of the cell population. Measurements were automatically collected every 15 min for 96 hours. Data were analyzed with the provided real-time cell analysis (RTCA) software. A similar protocol was applied to monitor the migration of cells transfected with an LNA Gapmer directed against a candidate lncRNA. HCT-116 cells were seeded 24 h post-transfection into the xCELLigence CIM-plate 16 (Roche) and 165 µl fresh medium containing 10% FBS (chemoattractant) or not (control) was added to the lower chamber of the CIM-plate 16. The upper chamber was filled with serum-free medium (50 µl/well) and the plate was incubated at 37° C under 5% CO2 for 1 h. Cells (80000 cells/well) were then added to each well of the upper chamber and cell migration was assessed at 30-min intervals for 48 hours at 37° C under 5% CO2. Upon migration, cells adhere to the surface of the filter electrode and increase the impedance, which is then used to derive a cell index reflecting the ability of the cell population to migrate.

### Protein extraction and western blot analyses

Whole-cell extracts were prepared in 250 µl or 500 µl (depending on the pellet size) of IPH lysis buffer (50 mM Tris-HCl pH 8, 150 mM NaCl, 5 mM ethylenediaminetetraacetic acid (EDTA), 0.5% NP40) supplemented with anti-protease cocktail (Promega). The suspensions were placed on ice for 30 minutes with periodic shaking and then centrifuged at 12,000 g for 10 min at 4° C. Supernatants were then collected and stored at −80° C until use. Supernatants were thawed on ice and added in appropriate proportions to 6X loading buffer (50 mM Tris-HCl pH 6.8, 2.5% v/v glycerol, 1% w/v SDS, 1% v/v beta-mercaptoethanol). Samples were then boiled at 95° C for 5 minutes. Migration was carried out in 7% acrylamide gel at 100 V for 80 minutes and then the proteins were transferred to a PVDF membrane (Millipore) at 100 V for 70 minutes. Membranes were incubated with blocking buffer (PBS, 1% Tween-20, 5% w/v non-fat dried milk) for 1 h at room temperature and primary antibodies were then added for overnight incubation at 4° C. Proteins were detected with West Femto Maximum Sensitivity Substrate (ThermoFisher). For ZEB1 we used a rabbit mAb (Cell Signaling Technology, 3396S) at 1/1000 dilution, for ACTB we used a mouse mAb (Sigma, A5316, also at 1/1000 dilution.

### Statistical analyses

Normal distribution of expression signals was assessed using the Shapiro.test function in the Rstudio software. We considered the distribution to be when the returned “Shapiro value” was greater than 0.05. Homoscedasticity was probed with the Bartlett test function and values were considered homoscedastic when the returned “Bartlett value” was greater than 0.05. As a few cases did not respect normal distribution or homoscedasticity we applied the Wilcoxon test (with the wilcox.test function, default parameters) to assess the significance of the observed differences. *P* values were then corrected for multi-testing with the p.adjust function of the Rstudio software, with the method parameter set at “BH” (Benjamini-Hochberg). Events were considered significant when the associated *p*-value or *q*-value (FDR) was smaller than 0.05. Association of the lncRNA level with the relapse-free survival period was assessed with the cox.zph function in Rstudio.

To identify significant differences in qPCR or western blot analyses, a *t*-test was applied to the observed measurements. Differences were considered significant when the associated *p*-value was smaller than 0.05.

## SUPPLEMENTARY MATERIALS FIGURES AND TABLES


















