# Supplementary material for: Comprehensive identification of long noncoding RNAs in colorectal cancer
Source: Oncotarget. 2018 Jun 12;9(45):27605–29. doi: 10.18632/oncotarget.25218 (PMC6021240; doi:10.18632/oncotarget.25218)
Supplement: Supplementary file 1 [file oncotarget-09-27605-s001.pdf]

Comprehensive identification of long noncoding RNAs in colorectal cancer

SUPPLEMENTARY MATERIALS

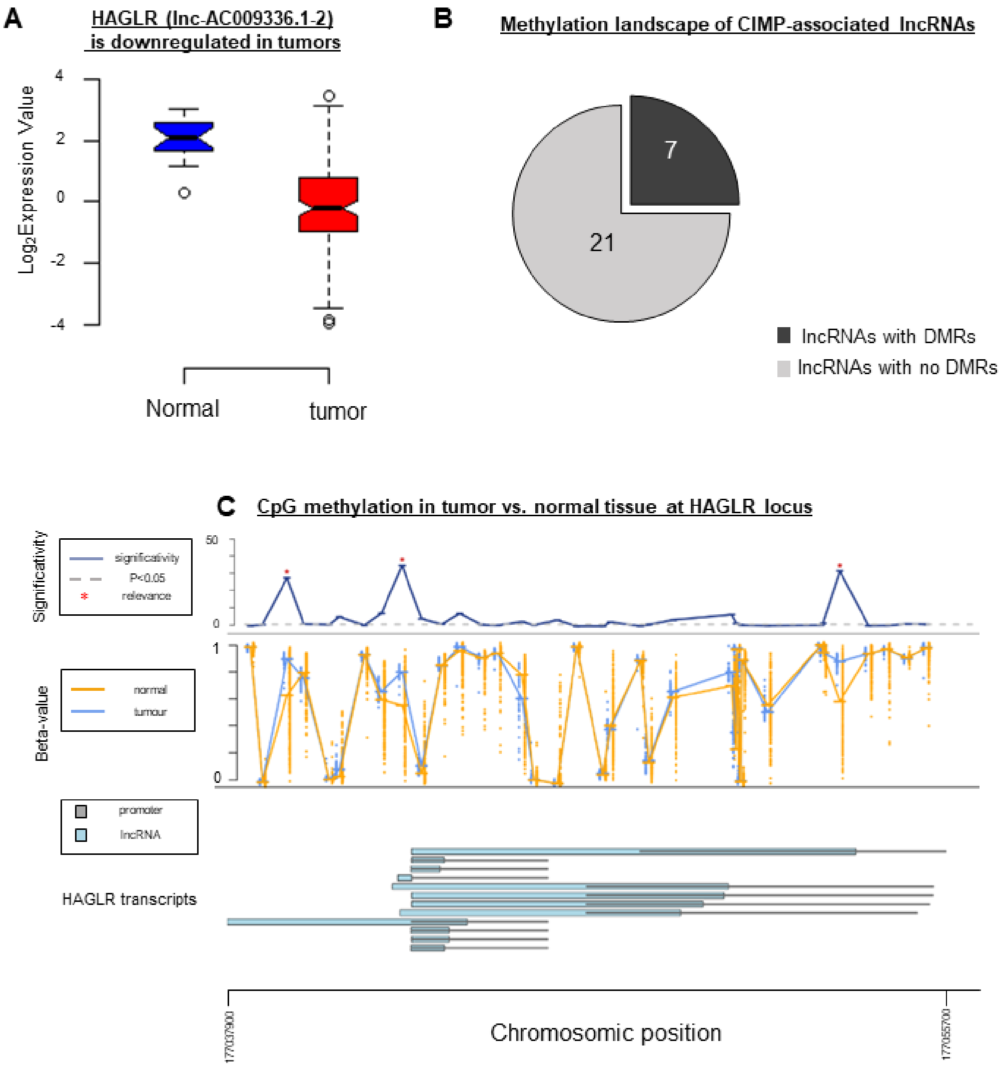

Supplementary Figure 1: CIMP-associated lncRNA levels and DNA methylation in tumors vs. normal tissues. (**A**) In the TCGA cohort, HAGLR is downregulated (FC = 0.21, FDR = 4.23E-12) in tumor (n = 155) vs. normal tissue samples (n = 19). Box plot description: the bold line is the median, the borders of the box are the first and third quartiles and the whiskers (error bars) are the most extreme expression values not greater than 1.5 times the interquartile range. The notches represent the 95% confidence interval. (**B**) Distribution of differentially methylated regions (DMRs) (Δ beta > 0.2, FDR < 0.05) among CIMP-associated lncRNAs in tumor vs. normal tissues. (**C**) Infinium 450k CpG methylation profiles (Beta-value) for colon adenocarcinoma primary tumors (n = 273, orange) and normal tissues (n = 38, blue) at the HAGLR transcription site. A probe is defined as located in the promoter of an lncRNA if the targeted cytosine is located between -2kb and +1kb from the transcription start site (TSS). A probe is considered differentially methylated if the difference between the two beta-values (Δ beta) exceeds 20%, $p < 0.05$. Top panel, significance of Δ beta for each CpG. Lower panel, beta-value profiles primary tumours and normal tissues (promoter region not drawn to scale).

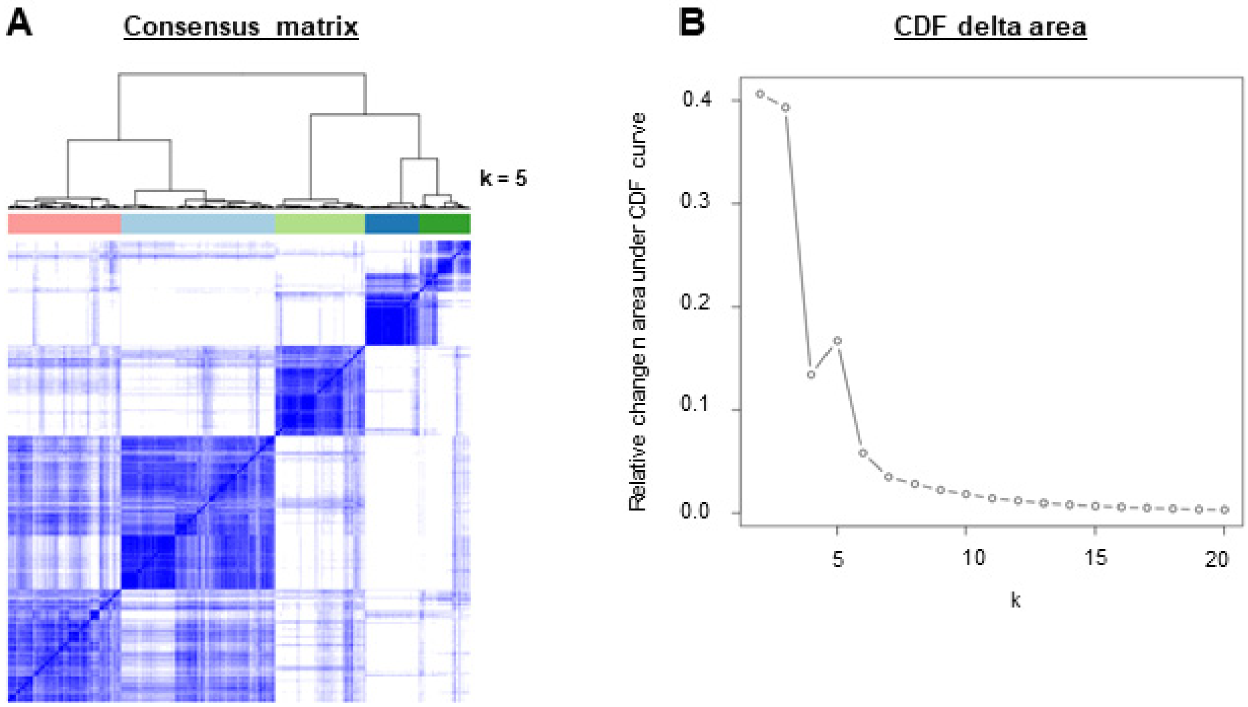

Supplementary Figure 2: Consensus clustering of CRC samples. The 566 tumors of the GSE39582 cohort were classified according to the expression profiles of the lncRNAs associated with tumor site, MMR, CIMP, or CIN status. (**A**) Consensus clustering matrix. (**B**) Delta area of the cumulative distribution function (CDF). This plot shows that six clusters is the optimal number as, a larger number of clusterd does not lead to an appreciable increase in the area under the CDF.

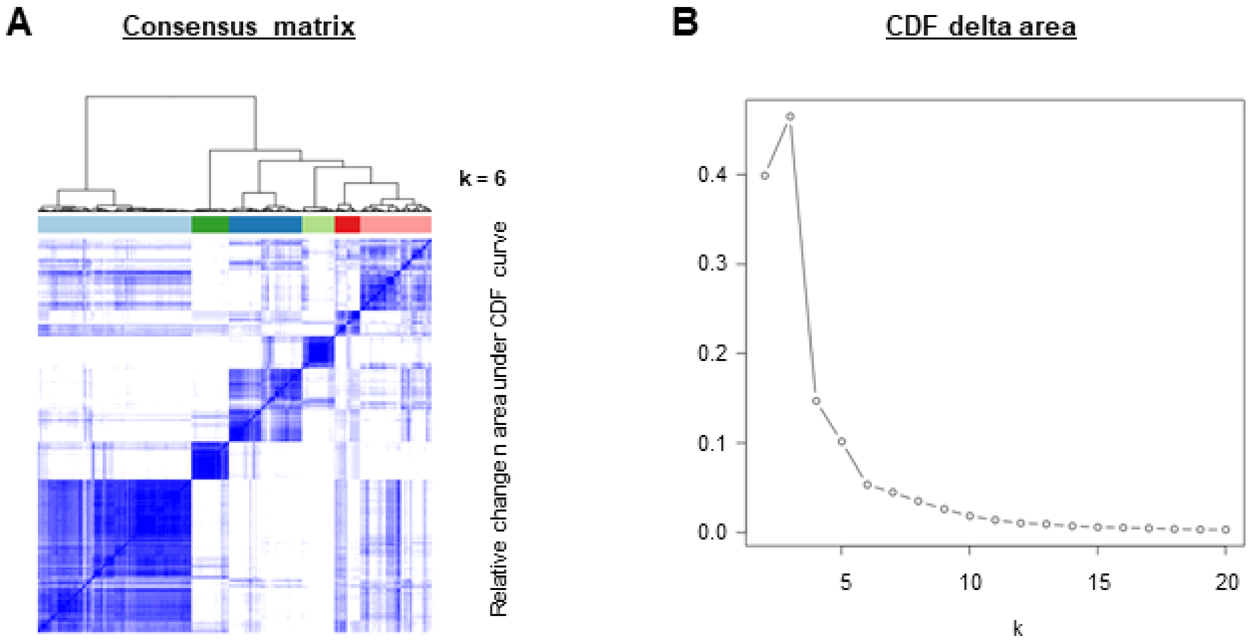

Supplementary Figure 3: Consensus clustering of CRC samples. The 566 tumors of the GSE39582 cohort were classified according to the expression profiles of the lncRNAs associated with CRC subtypes. (**A**) Consensus clustering matrix. (**B**) Delta area of the cumulative distribution function. This plot shows that six clusters is the optimal number, as a larger number of clusters does not lead to an appreciable increase in the area under the CDF.

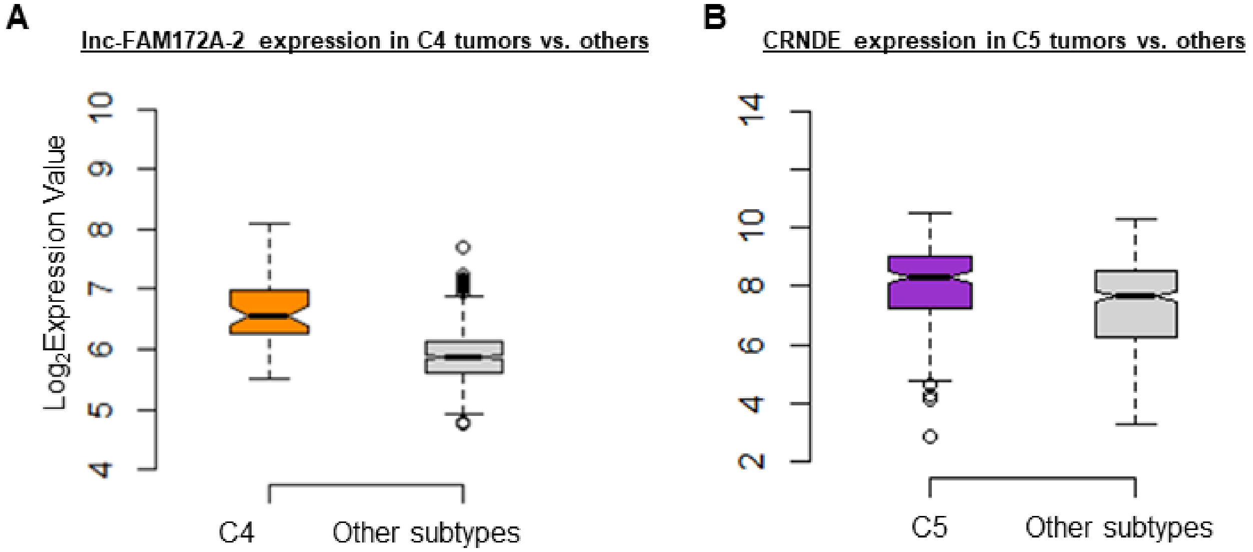

Supplementary Figure 4: CRC subtypes display differential regulation of lncRNAs. (**A**) lnc-FAM172A-2 was upregulated (FC = 1.6, FDR = 3.24E-12) in the C4 subtype. (**B**) CRNDE (aka lnc-IRX3-4) exhibited higher levels in the C5 subtype (FC = 1.54, FDR = 0.00034). *P*-values were assessed with the *t*-test and corrected for multitesting with the Benjamini-Hochberg method. Box plot description: the bold line is the median, the borders of the box are the first and third quartiles, and the whiskers (error bars) are the most extreme expression values not greater than 1.5 times the interquartile range. The notches represent the 95% confidence interval.

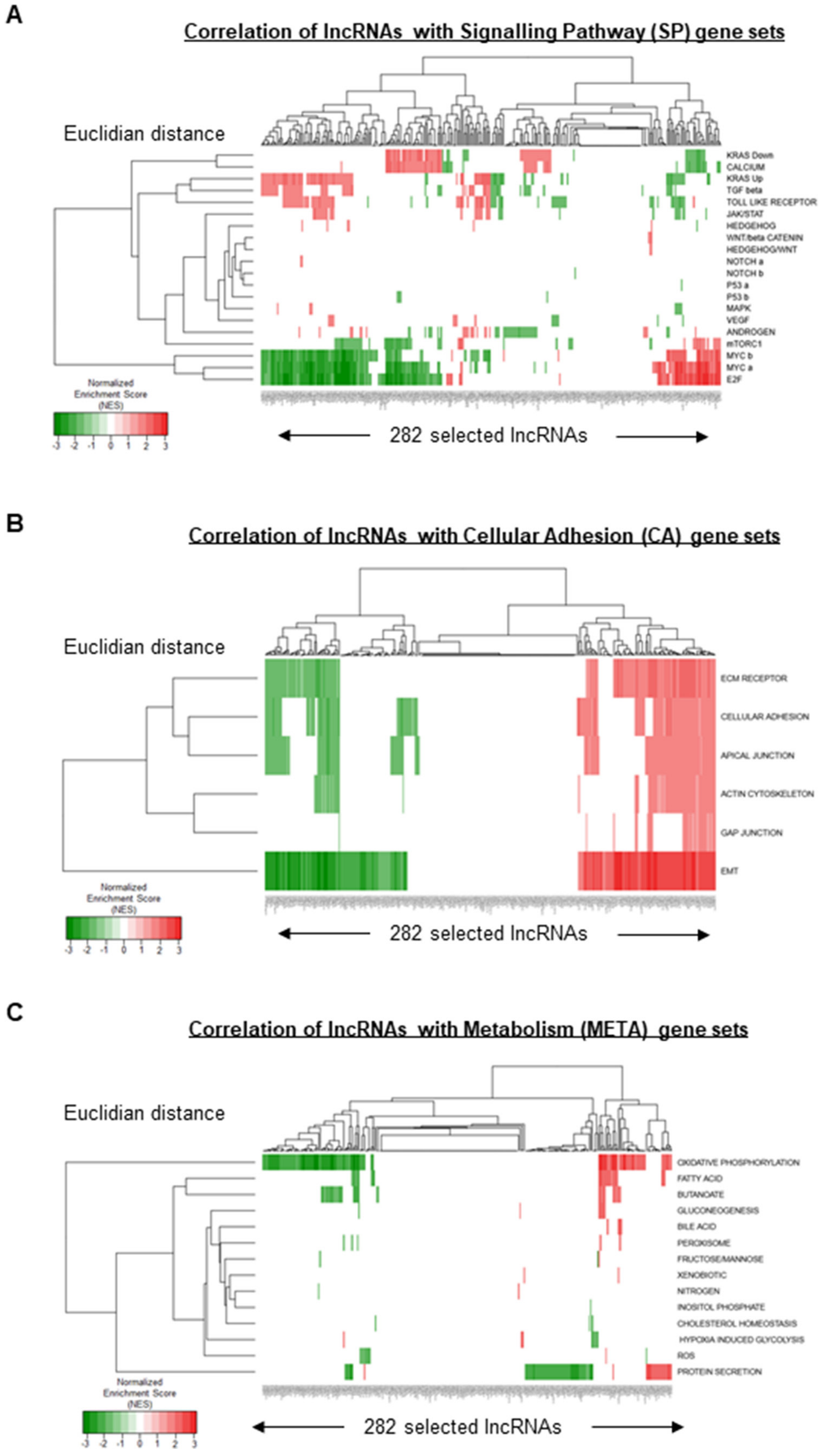

A
Correlation of lncRNAs with Signalling Pathway (SP) gene sets
Euclidian distance
KRAS Down
CALCIUM
KRAS Up
TGF beta
TOLL LIKE RECEPTOR
JAK/STAT
HEDGEHOG
WNT/beta CATENIN
HEDGEHOG/WNT
NOTCH a
NOTCH b
P53 a
P53 b
MAPK
VEGF
ANDROGEN
mTORC1
MYC b
MYC a
E2F
Normalized
Enrichment Score
(NES)
-3 -2 -1 0 1 2 3
282 selected lncRNAs
B
Correlation of lncRNAs with Cellular Adhesion (CA) gene sets
Euclidian distance
ECM RECEPTOR
CELLULAR ADHESION
APICAL JUNCTION
ACTIN CYTOSKELETON
GAP JUNCTION
EMT
Normalized
Enrichment Score
(NES)
-3 -2 -1 0 1 2 3
282 selected lncRNAs
C
Correlation of lncRNAs with Metabolism (META) gene sets
Euclidian distance
OXIDATIVE PHOSPHORYLATION
FATTY ACID
BUTANOATE
GLUCONEOGENESIS
BILE ACID
PEROXISOME
FRUCTOSE/MANNOSE
XENOBIOTIC
NITROGEN
INOSITOL PHOSPHATE
CHOLESTEROL HOMEOSTASIS
HYPOXIA INDUCED GLYCOLYSIS
ROS
PROTEIN SECRETION
Normalized
Enrichment Score
(NES)
-3 -2 -1 0 1 2 3
282 selected lncRNAs

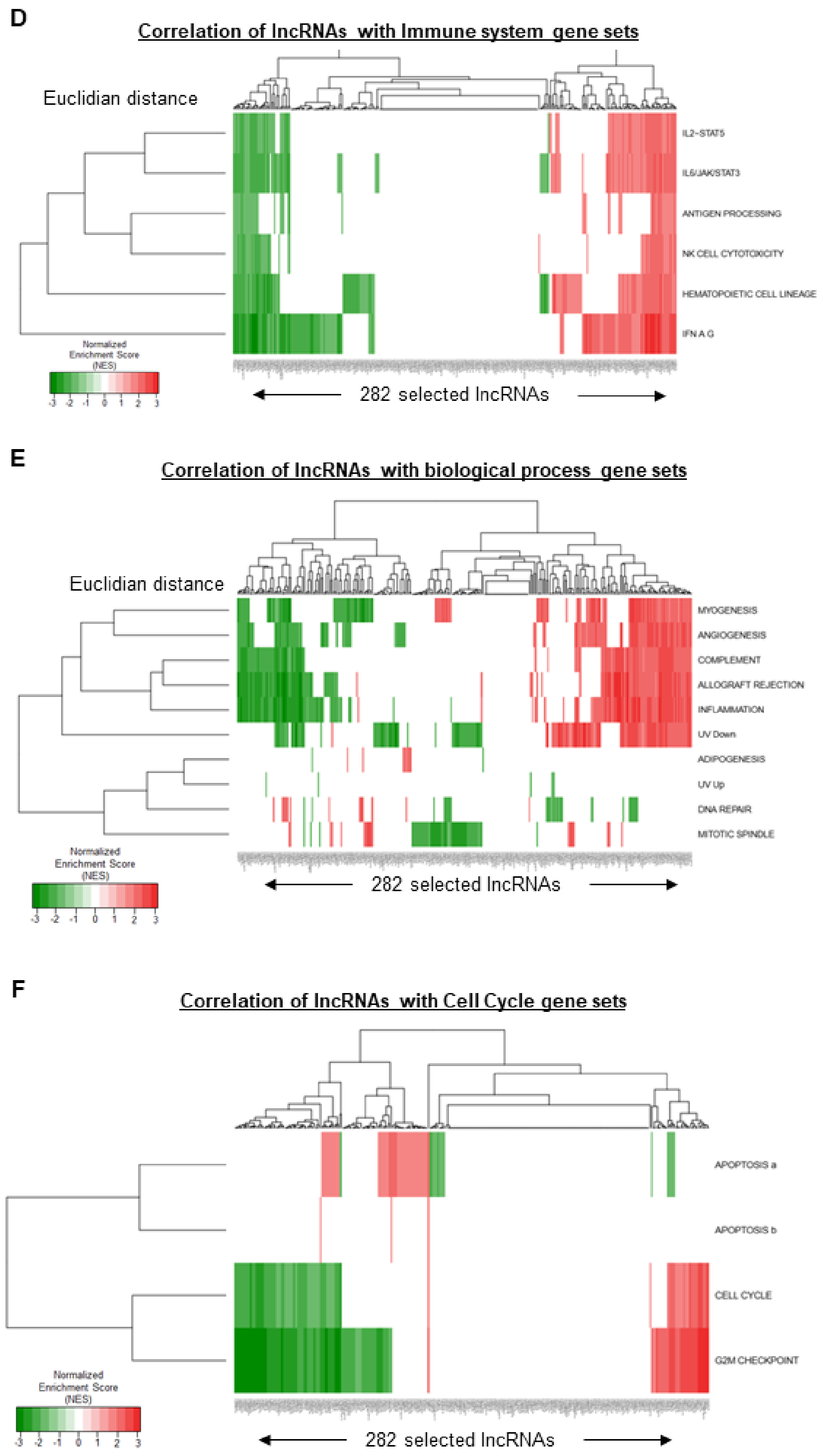

Supplementary Figure 5: Functional characterization: Normalized Enrichment Scores (NESs) of 282 selected lncRNAs. NESs reflect correlations between expression levels of lncRNA genes and those of genes (grouped in gene sets) involved in a particular function or process. NES heatmap illustrating pathways (or processes) whose activation (green) or inhibition (red) correlates with expression one or several of the 282 lncRNAs selected for their association with a CRC heterogeneity marker (CIMP status, BRAF mutation status…) in 566 CRC samples. (**A**) NES heatmap representing predicted associations of lncRNAs with "signaling pathway" gene sets. (**B**) NES heatmap for "cell adhesion" gene sets. (**C**) NES heatmap for "metabolic pathway" gene sets. (**D**) NES heatmap for "immune system" gene sets. (**E**) NES heatmap for "biological process" gene sets. (**F**) NES heatmap for "cell cycle" gene sets. All NESs were statistically significant (FWER < 0.05), see methods.

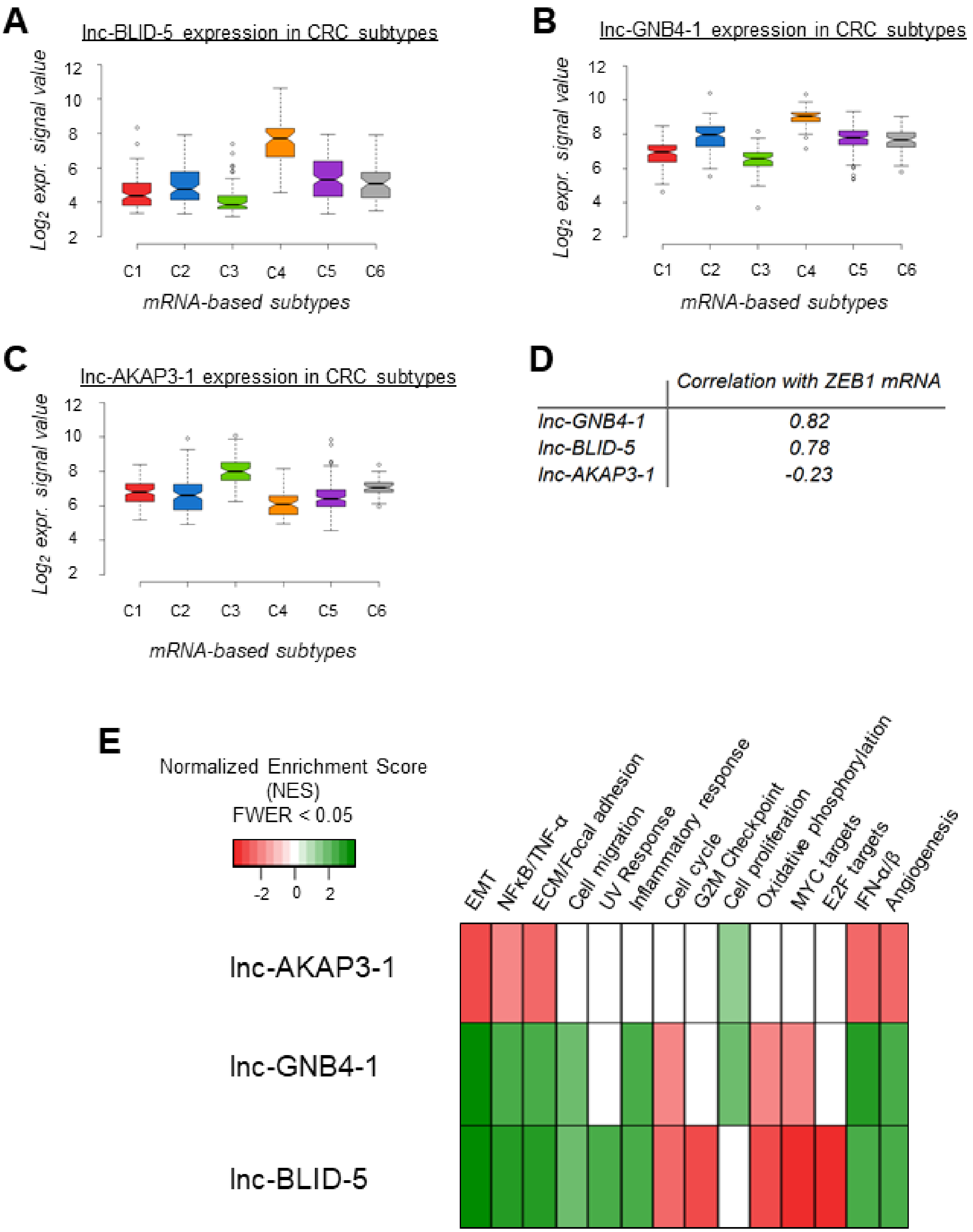

Supplementary Figure 6: Expression patterns of lncRNA genes selected for in vitro experimentation. (**A**) Expression levels of the lnc-BLID-5 gene across the six CRC subtypes. (**B**) Expression levels of the lnc-GNB4-1 gene across the six CRC subtypes. (**C**) Expression levels of the lnc-AKAP3-1 gene across the six CRC subtypes. Subtypes were previously defined on the basis of mRNA-level gene expression [12]. Box plot description: the bold line is the median, the borders of each box are the first and third quartiles, and the whiskers (error bars) are the most extreme expression values not greater than 1.5 times the interquartile range. The notches represent the 95% confidence interval. (**D**) Correlation coefficients between the expression levels of three candidate lncRNA genes and mRNA-level expression of the gene encoding the ZEB1 transcription factor. (**E**) Normalized Enrichment Score (NES) heatmap, describing the directions and intensities of associations of candidate lncRNAs with 14 gene sets. All NESs were statistically significant (FWER < 0.05).

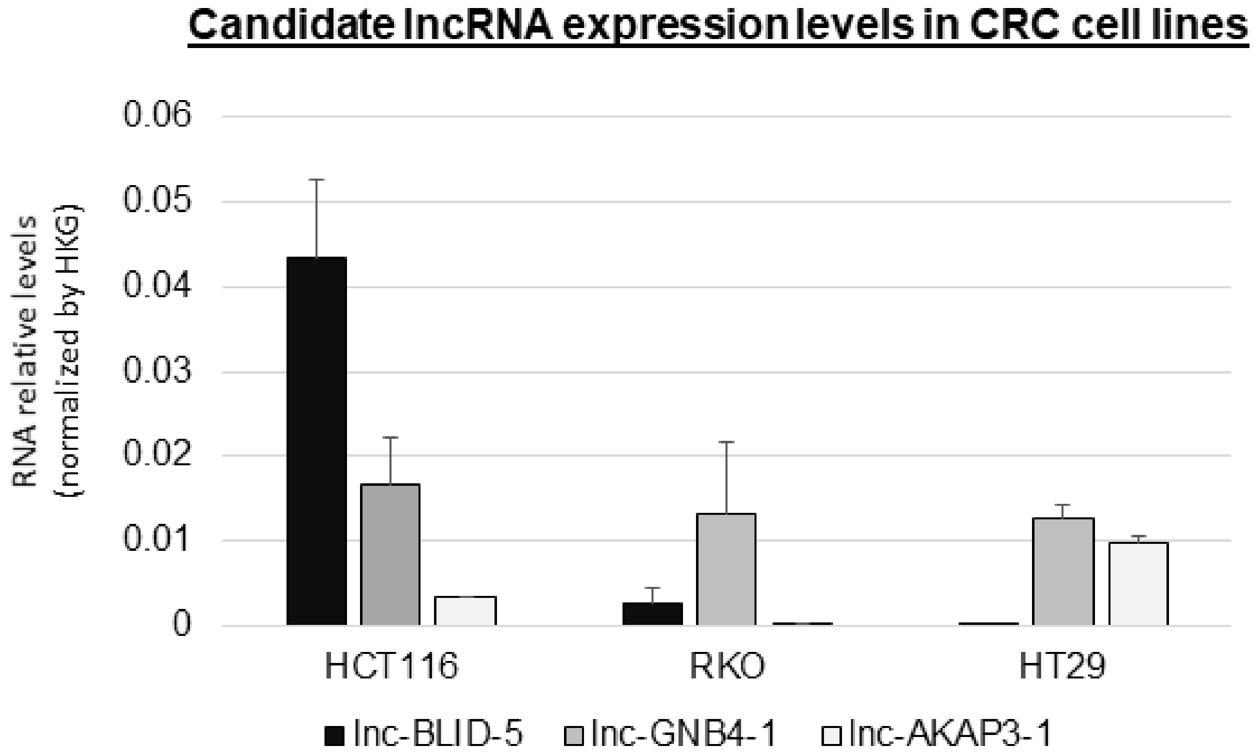

Supplementary Figure 7: Candidate lncRNAs are differentially expressed in CRC cell lines. RT-qPCR results, showing the relative expression levels of lnc-BLID-5 (black), lnc-GNB4-1 (gray) and lnc-AKAP3-1 (light gray) in HCT-116, RKO and HT-29 cells. Normalized with respect to the housekeeping genes (HKG) HPRT and SDHA. Data are presented as averages of three independent experiments. Error bars represent standard deviations.

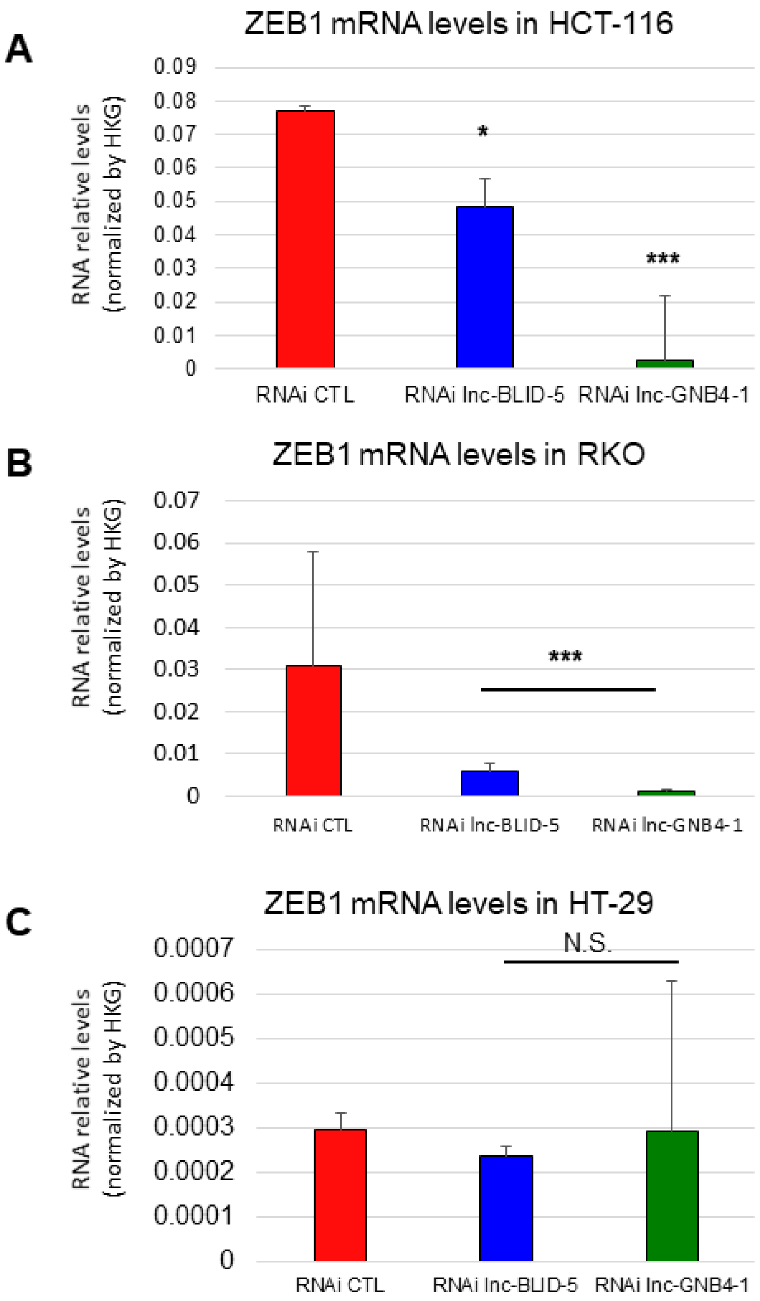

Supplementary Figure 8: Depletion of lnc-BLID-5 and lnc-GNB4-1 affect ZEB1 mRNA levels in cells. (**A**) RNA interference (RNAi) mediated knock-down of lnc-BLID-5 (blue) and lnc-GNB4-1 (dark green) (see material and methods) in HCT-116 cells. (**B** and **C**) Same as A for RKO and HT-29 cells respectively. Both lncRNA appear to favour the transcription of ZEB1 proteins as their depletion significantly decreased ZEB1 mRNA levels. Data represent the average of three independent experiments, errors bars represent standard deviation. Significant differences were evaluated with a two-tailed paired *t*-test ($p < 0.05 = {}^{*}p < 0.01 = {}^{**}p < 0.001 = {}^{***}$).

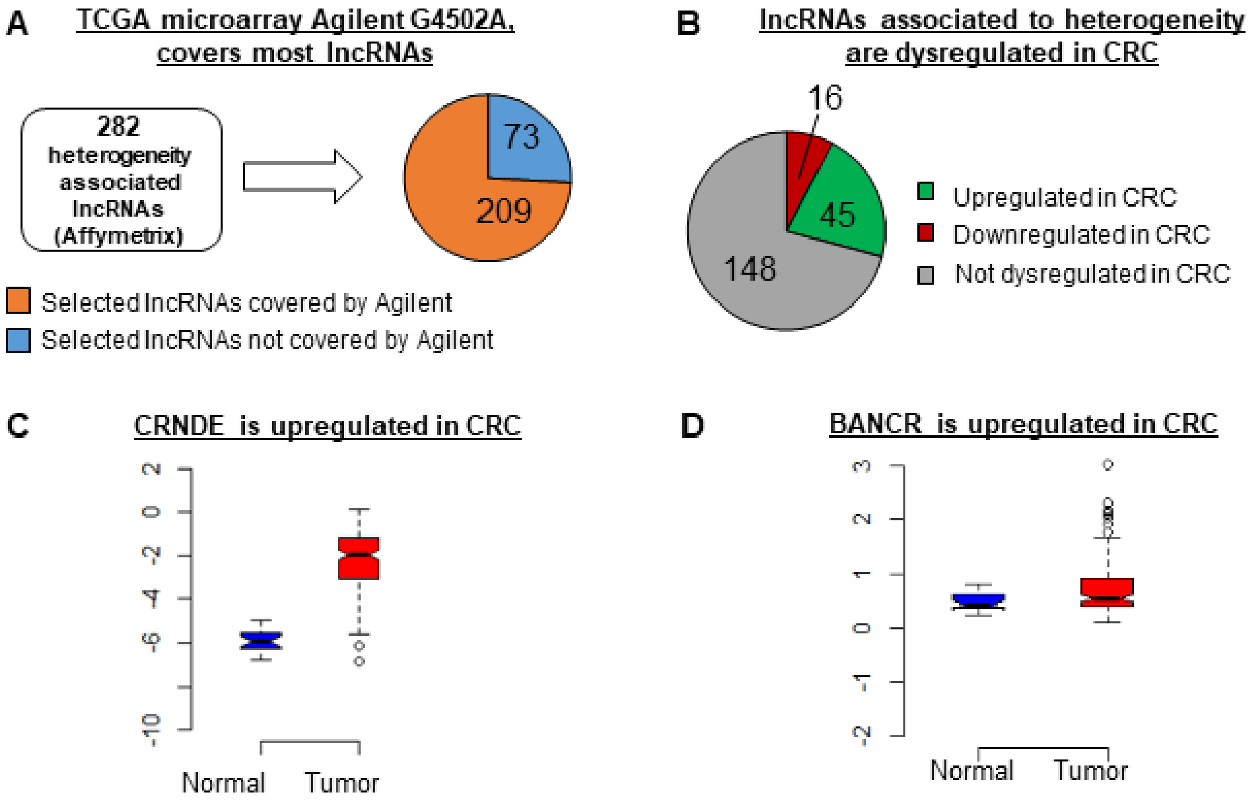

Supplementary Figure 9: Heterogeneity associated lncRNAs are dysregulated in CRC compared to normal tissues. (**A**) The TCGA microarray (Agilent) covers 209 out of the 282 selected lncRNAs which were present on the Affymetrix microarray used in the GSE39582 cohort and used in this study. (**B**) Out of the 209 lncRNAs which were covered by the TCGA microarray, we observed significant upregulation (16 lncRNAs) and downregulation (45 lncRNAs) in primary tumor samples. (**C**) CRNDE (lnc-IRX3-4) was upregulated (FC = 12.8, FDR = 5.25E-12) in the tumor samples of the TCGA cohort, as previously reported [32]. (**D**) BANCR (lnc-APBA1-3) was upregulated (FC = 1.34, FDR = 0.025) in tumor samples in the GSE39582 cohort (*n* = 566) as previously reported [18].

Supplementary Table 1: Description of the GSE39582 cohort

Affymetrix gene expression microarray HG U133 PLUS 2 (*n* = 566) (GSE39582, Marisa *et al*.)
**Mismatch Repair Machinery (MMR) status**
Deficient (dMMR) = 75
Proficent (pMMR) = 444
NA = 47
**Chromosome stability (CIN) status**
CIN high = 110
CIN low = 354
NA = 102
**CpG Island Methylator Phenotype (CIMP) status**
CIMP + (positive) = 91
CIMP - (negative) = 405
NA = 70
**BRAF mutations**
BRAF mutated = 51
BRAF wild-type = 461
NA = 49
**KRAS mutations**
KRAS mutated = 217
KRAS wild-type = 328
NA = 21
P53 mutations
P53 mutated = 190
P53 wild-type = 161
NA = 215
**mRNA expression based CRC subtypes (Marisa *et al*.)**
C1 (CIN high, immune down) = 116
C2 (dMMR) = 104
C3 (KRAS mutation) = 75
C4 (Cancer Stem Cell, EMT ‘high’) = 59
C5 (CIN high, WNT up) = 152
C6 (CIN high, normal-like) = 60
**Agilent gene expression microarray (G4502A)**
Tumor tissue samples = 155
Normal tissue samples = 19
**Infinium 450 k DNA methylation array**
Tumor tissue samples = 273
Normal tissue samples = 38

Supplementary Table 2: Affymetrix array reannotation. See Supplementary_Table_2

Supplementary Table 3: Information on the 282 unique lncRNAs. See Supplementary_Table_3

Supplementary Table 4: Dysregulation of lncRNAs in tumor vs. normal colorectal tissue (TCGA cohort). See Supplementary_Table_4

Supplementary Table 5: Clinical annotation of samples from the GSE39582 cohort. See Supplementary_Table_5

Supplementary Table 6: Univariate Cox analysis details. See Supplementary_Table_6

Supplementary Table 7: Multiivariate Cox analysis details. See Supplementary_Table_7

Supplementary Table 8: Normalized Enrichment Scores (NES). See Supplementary_Table_8

Supplementary Table 9: Genesets. See Supplementary_Table_9
